# Rapid Evolution of Enormous, Multichromosomal Genomes in Flowering Plant Mitochondria with Exceptionally High Mutation Rates

**DOI:** 10.1371/journal.pbio.1001241

**Published:** 2012-01-17

**Authors:** Daniel B. Sloan, Andrew J. Alverson, John P. Chuckalovcak, Martin Wu, David E. McCauley, Jeffrey D. Palmer, Douglas R. Taylor

**Affiliations:** 1 Department of Biology, University of Virginia, Charlottesville, Virginia, United States of America; 2 Department of Biological Sciences, University of Arkansas, Fayetteville, Arkansas, United States of America; 3 Department of Biological Sciences, Vanderbilt University, Nashville, Tennessee, United States of America; 4 Department of Biology, Indiana University, Bloomington, Indiana, United States of America; Dalhousie University, Canada

## Abstract

Genome size and complexity vary tremendously among eukaryotic species and their organelles. Comparisons across deeply divergent eukaryotic lineages have suggested that variation in mutation rates may explain this diversity, with increased mutational burdens favoring reduced genome size and complexity. The discovery that mitochondrial mutation rates can differ by orders of magnitude among closely related angiosperm species presents a unique opportunity to test this hypothesis. We sequenced the mitochondrial genomes from two species in the angiosperm genus *Silene* with recent and dramatic accelerations in their mitochondrial mutation rates. Contrary to theoretical predictions, these genomes have experienced a massive proliferation of noncoding content. At 6.7 and 11.3 Mb, they are by far the largest known mitochondrial genomes, larger than most bacterial genomes and even some nuclear genomes. In contrast, two slowly evolving *Silene* mitochondrial genomes are smaller than average for angiosperms. Consequently, this genus captures approximately 98% of known variation in organelle genome size. The expanded genomes reveal several architectural changes, including the evolution of complex multichromosomal structures (with 59 and 128 circular-mapping chromosomes, ranging in size from 44 to 192 kb). They also exhibit a substantial reduction in recombination and gene conversion activity as measured by the relative frequency of alternative genome conformations and the level of sequence divergence between repeat copies. The evolution of mutation rate, genome size, and chromosome structure can therefore be extremely rapid and interrelated in ways not predicted by current evolutionary theories. Our results raise the hypothesis that changes in recombinational processes, including gene conversion, may be a central force driving the evolution of both mutation rate and genome structure.

## Abstract 2

A pair of species within the genus *Silene* have evolved the largest known mitochondrial genomes, coinciding with extreme changes in mutation rate, recombination activity, and genome structure.

## Author Summary

A fundamental challenge in evolutionary biology is to explain why organisms exhibit dramatic variation in genome size and complexity. One hypothesis predicts that high rates of mutation in DNA sequence create selection against large and complex genomes, which are more susceptible to mutational disruption. Species of flowering plants in the genus *Silene* vary by approximately 100-fold in the rates of mutation in their mitochondrial DNA, providing an excellent opportunity to test the predicted effects of high mutation rates on genome evolution. Contrary to expectation, *Silene* species with elevated mutation rates have experienced dramatic expansions in mitochondrial genome size compared to their slowly evolving relatives, resulting in the largest known mitochondrial genomes. In addition to the increases in size and mutation rate, these genomes also reveal a history of rapid change in genome structure. They have been fragmented into dozens of chromosomes and appear to have experienced major reductions in recombination activity. All of these changes have occurred in just the past few million years. This mitochondrial genome diversity within the genus *Silene* provides a striking example of rapid genomic change and raises new hypotheses regarding the relationship between mutation rate and genome evolution.

## Introduction

Explaining the origins of variation in genome size and complexity has become the defining challenge for the field of molecular evolution in the genomic era. Historically, numerous evolutionary models have been developed, involving mechanisms such as insertion and deletion (indel) bias [1],[2], selfish element proliferation [3],[4], and natural selection on cell size [5], replication rate [6], and evolvability [7]. In recent years, a body of theory known as the mutational burden hypothesis (MBH) has emerged as a potentially unifying explanatory framework rooted in the principles of population genetics and the basic evolutionary processes of mutation and genetic drift [8],[9]. The MBH posits that noncoding elements are generally deleterious but proliferate nonadaptively when small effective population sizes reduce the effectiveness of selection relative to genetic drift, offering an explanation for why noncoding sequences are so abundant in large multicellular eukaryotes. This hypothesis is based on the idea that noncoding elements impose a selective cost associated with the increased chance of mutations disrupting an essential genome function (e.g., alteration of a conserved sequence required for intron splicing) or generating a novel deleterious feature (e.g., an improper transcription-factor binding site in an intergenic region). The MBH has potentially sweeping explanatory power, but some of its tenets are controversial [10]–[13], and its generality as a mechanism of genome evolution remains uncertain [14]–[21].

Mitochondrial genomes display striking diversity in size and complexity [22],[23], reflecting patterns of variation in genome architecture observed more broadly across the tree of life [9],[24]. For example, in contrast to the small (typically 14–20 kb) and streamlined genomes found in most animal mitochondria [25], seed plant mitochondrial genomes are very large (200–2,900 kb), containing introns and abundant intergenic sequences [26]–[28]. Plant mitochondrial genomes are also typically characterized by extremely low point mutation rates, further distinguishing them from their fast-evolving animal counterparts [29]–[31]. The observed disparity in mitochondrial mutation rates across eukaryotes motivated the hypothesis that mutation rates are a major determinant of variation in organelle genome architecture [32]. This argument is a direct extension of the MBH and is based on the premise that the probability of mutational disruption of noncoding elements (which is equivalent to the selective cost associated with maintaining those elements) is directly proportional to the mutation rate. Therefore, genomes with elevated mutation rates are predicted to experience more intense selection for genomic reduction [32].

The discovery that some angiosperms have greatly accelerated mitochondrial mutation rates, sometimes orders of magnitude greater than closely related species [33]–[35], presents an opportunity to test the prediction that high mutation rate environments select for reduced and streamlined genomes. In particular, several species in the genus *Silene* (Caryophyllaceae) have experienced dramatic increases in mitochondrial mutation rates within just the last 5–10 Myr, while other members of this genus have maintained their ancestrally low rates [35]–[37].

We compared complete mitochondrial genome sequences from four *Silene* species with very different mutation rates and found that accelerated mutation rates have indeed been associated with dramatic changes in genome size and complexity. However, the direction of these changes is not always consistent with the predictions from existing theory. We discuss the implications of the unprecedented mitochondrial genome diversity found within *Silene* and possible alternative explanations for the rapid genome evolution in this genus.

## Results

### Variation in Mitochondrial Substitution and Indel Rates within *Silene*

**Figure 1:**
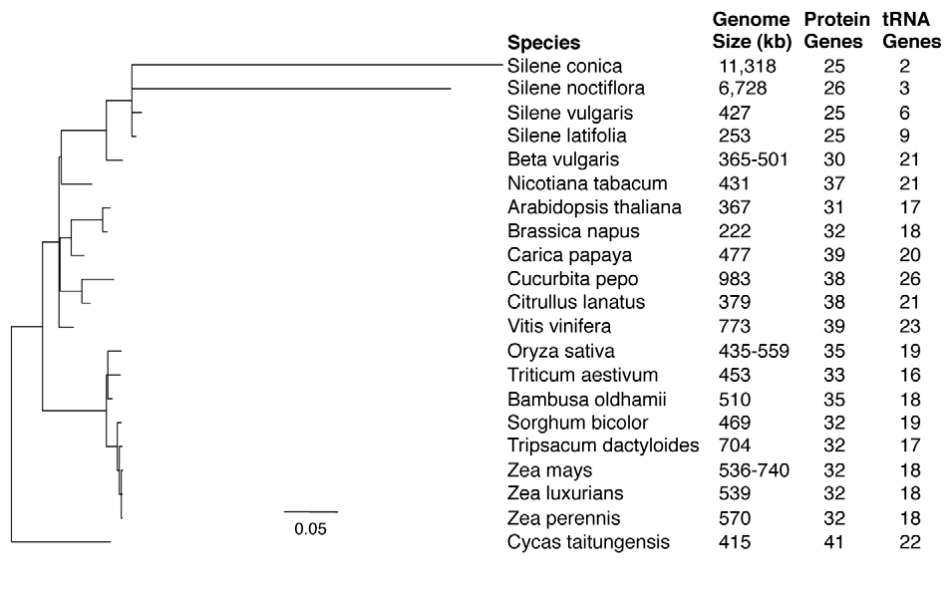
Sequence divergence, genome size, and gene content in seed plant mitochondria. Branch lengths are scaled to the number of synonymous nucleotide substitution per site (*d*_S_) on the basis of an analysis of all shared protein genes. Genome size ranges are reported for species with multiple sequences available. Gene counts exclude duplicates and putative pseudogenes.

**Figure 2:**
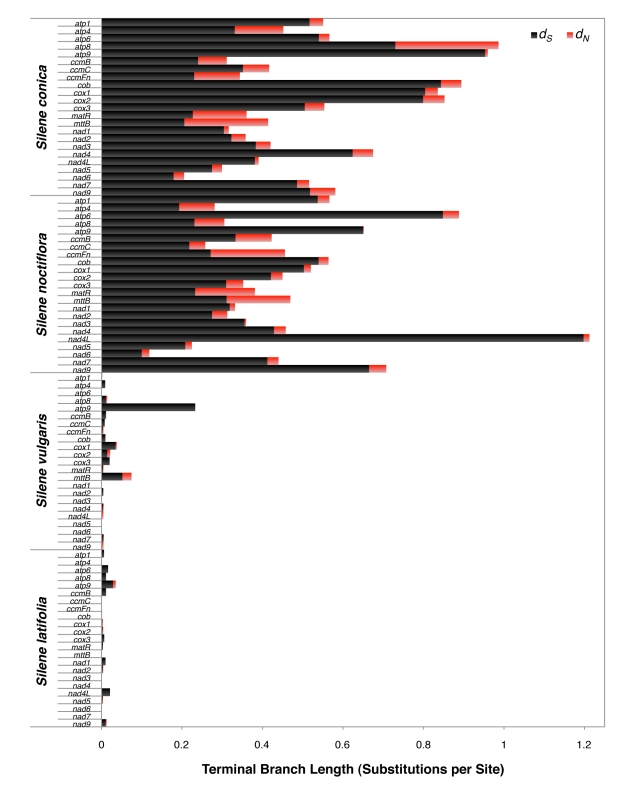
Levels of synonymous (*d*_S_) and nonsynonymous (*d*_N_) sequence divergence in terms of substitutions per site for protein genes in *Silene* mitochondrial genomes. Estimates were generated using *B. vulgaris* and *A. thaliana* as outgroups.

**Figure 3:**
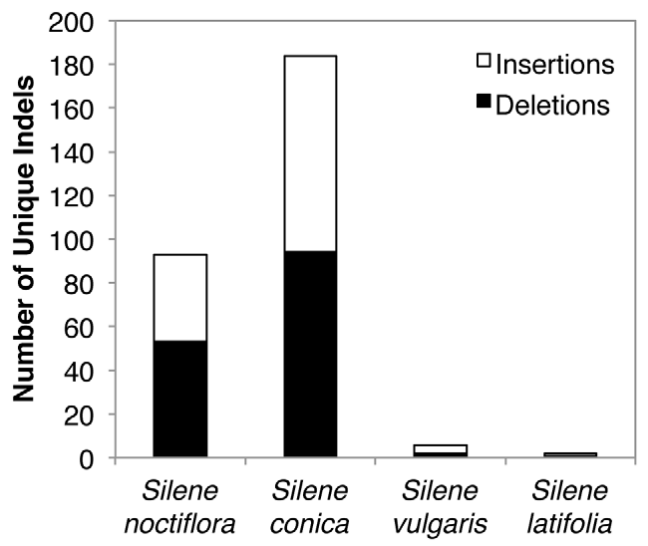
Number of indels in mitochondrial protein genes and introns that are unique to each of the four *Silene* species.

Sequencing of purified mitochondrial DNA (mtDNA) from three *Silene* species generated complete genome assemblies for *S. noctiflora* and *S. vulgaris* and a high quality draft assembly for *S. conica*. We also included the previously published mitochondrial genome of *S. latifolia* in our analyses [38]. The genomic data extend previous results [35]–[37] by showing that *S. noctiflora* and *S. conica* have experienced massive accelerations in nucleotide substitution rates (Figure 1) across all protein genes (Figure 2) with correlated increases in the frequency of both insertions and deletions (Figure 3).

### Massive Mitochondrial Genome Expansion in High Mutation-Rate Species

Contrary to the prediction of genomic streamlining in response to high mutation rate, the fast-evolving mitochondrial genomes of *S. noctiflora* and *S. conica* have experienced unprecedented expansions, resulting in sizes of 6.7 Mb and 11.3 Mb, respectively. In contrast, the more typical slowly evolving mitochondrial genomes of *S. vulgaris* (0.43 Mb) and particularly *S. latifolia* (0.25 Mb) are on the lower end of the angiosperm size range. Thus, *Silene* mitochondrial genomes have diverged more than 40-fold in size in just the past few million years.

**Figure 4:**
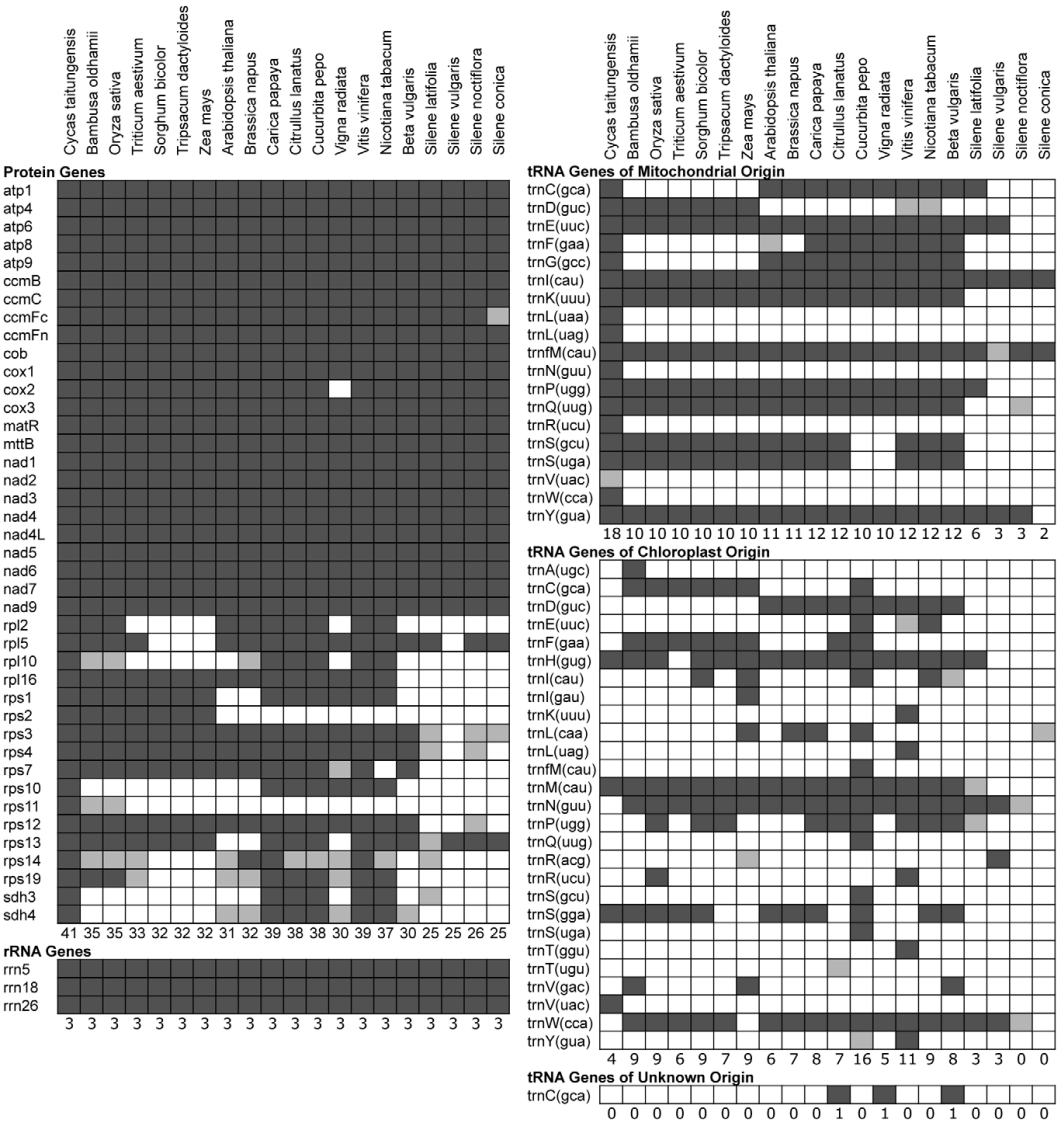
Protein and RNA gene content in sequenced seed plant mitochondrial genomes. Dark shading indicates the presence of an intact reading frame or folding structure, whereas light shading indicates the presence of only a putative pseudogene. The numbers at the bottom of each group indicate the total number of intact genes for that species. Note that the *ccmFc* gene, which is universally present in all other seed plants surveyed to date [104], is classified as a pseudogene in *S. conica*. It has experienced numerous structural mutations in this lineage, including multiple frame shifts in the second exon that introduce premature stop codons. However, cDNA sequencing confirmed that this gene is transcribed, spliced, and RNA edited in *S. conica* (unpublished data), so it is possible that the gene is still functional in its truncated form. In some cases, the presence of an intact gene may not indicate functionality. This is particularly true for tRNA genes embedded within recently transferred regions of plastid DNA [20],[105]. For example, the *trnN(guu)* and *trnR(acg)* genes in *S. vulgaris* may not be functional, as they are within a 2.6-kb region that appears to have been recently transferred from the plastid genome (on the basis of its perfect sequence identity with the exception of a single 18-bp deletion). These two tRNA genes are not orthologous to the plastid-derived copies of *trnN(guu)* and *trnR(acg)* in other seed plant mitochondria. Intron-containing plastid-derived tRNA genes such as *trnA(ugc)* in *Bambusa*, *trnV(uac)* in *Cycas*, *trnK(uuu)* in *Vitis*, and *trnI(gau)* in *Zea* are also unlikely to be functional. In *Cycas*, the *trnL(uaa)*, *trnP(ugg)*, *trnQ(uug)*, *trnR(ucu)*, and *trnV(uac)-* Ψ genes are classified on the basis of sequence homology to other land plant tRNAs even though their genomically encoded anticodons differ (CAA, CGG, CUG, CCU, and CAC, respectively). It is possible that these anticodons undergo C-to-U RNA editing to restore the ancestral codon as has been observed in other vascular plants [106],[107]. Plastid-derived tRNAs with substitutions in their anticodons, such as *Citrullus trnT(ugu)* and *Silene latifolia trnP(ugg)*, are also classified (as pseudogenes) on the basis of homology.

The genomic expansion in *S. noctiflora* and *S. conica* does not reflect detectable increases in gene or intron content. Although these genomes contain duplicate copies of some genes (particularly rRNA genes; Table S1), they possess fewer unique genes than other angiosperm mitochondrial genomes (Figures 1 and 4). Notably, the *S. conica* and *S. noctiflora* mitochondrial genomes contain only two or three identifiable tRNA genes, which is far fewer than most angiosperms and even less than the already reduced tRNA gene content of *S. latifolia* and *S. vulgaris* (Figures 1 and 4) [38]. The four *Silene* genomes have nearly identical sets of introns (Table 1). With the exception of additional intron copies associated with gene duplications, there were no intron gains among the four *Silene* species and only one observed intron loss (the third intron of *nad4* in *S. noctiflora*). Interestingly, in contrast to the overall pattern of genome expansion in *S. noctiflora* and *S. conica*, average intron lengths in the expanded *S. noctiflora* and *S. conica* genomes are actually ∼10%–15% shorter than in their congeners (Figure S1).

**Table 1:** Summary of four *Silene* mitochondrial genomes.

Genome Characteristics	*S. latifolia*	*S. vulgaris*	*S. noctiflora*	*S. conica*
**Genome size in kb**	253	427	6,728	11,318
**Circular chromosomes**	1	4	59	128+
**Percent G+C content**	42.6	41.8	40.8	43.1
**Protein genes** a	25	25	26	25
**tRNA genes** a	9	6	3	2
Native	6	3	3	2
Plastid-derived	3	3^b^	0	0
**rRNA genes** a	3	3	3	3
**Introns** a	19	19	18	19
*cis*-spliced	13	13	12	13
*trans*-spliced	6	6	6	6
**Genic gontent in kb (percent coverage)** a	51 (20.3)	48 (11.2)	72 (1.1)	77 (0.7)
Exonic	34 (13.6)	31 (7.2)	58 (0.9)	57 (0.5)
Intronic^c^	17 (6.7)	17 (4.0)	14 (0.2)	20 (0.2)
**Intergenic content in kb (percent coverage)**	202 (79.7)	379 (88.8)	6,656 (98.9)	11,241 (99.3)
Plastid-derived	2 (1.0)	10 (2.3)	17 (0.3)	35 (0.3)
Conserved with other plant mtDNA^d^	95 (37.7)	73 (17.0)	843 (12.5)	834 (7.4)
Conserved with GenBank nr/nt^e^	5 (2.0)	3 (0.7)	20 (0.3)	16 (0.1)
Uncharacterized	99 (39.0)	294 (68.9)	5,776 (85.9)	10,356 (91.5)
**Repetitive content in kb (percent coverage)**	17 (6.7)	80 (18.8)	735 (10.9)	4,621 (40.8)
Large repeats: >1 kb	12 (4.9)	57 (13.3)	110 (1.6)	1,121 (9.9)
Small repeats: ≤1 kb	5 (1.8)	23 (5.5)	625 (9.3)	3,500 (30.9)
**RNA editing sites**	287	271^f^	189	182^f^
**Non-Syn. substitution rate (×10^−9^/y)**	0.08	0.35	8.90	9.98
**Syn. substitution rate (×10^−9^/y)**	0.70	1.60	58.17	68.22
***d*** **_N_/** ***d*** **_S_**	0.12	0.22	0.15	0.15

a Duplicate genes/introns are included in length and coverage statistics but excluded from reported counts.

b Two of the *S. vulgaris* plastid-derived tRNA genes may not be functional (Figure 4).

c Intron lengths only include *cis*-spliced introns.

d Excludes regions of plastid-origin.

e Excludes regions of plastid-origin and regions conserved in other plant mitochondrial genomes.

f Predicted.

Intergenic sequences account for 99% of the bloated mitochondrial genomes in *S. noctiflora* and *S. conica*. As in other vascular plants [28],[39], the intergenic regions of all four *Silene* mitochondrial genomes contain sequences of both nuclear and plastid (chloroplast) origin. Although the expanded mitochondrial genomes of *S. noctiflora* and *S. conica* contain more of this “promiscuous” DNA than their smaller *Silene* counterparts (Table 1), contributions from these sources do not scale proportionally with the increases in genome size and constitute less than 1% of the intergenic content in both species (Table 1). A larger fraction of the intergenic regions in each of these two genomes exhibit similarity to sequences in other plant mitochondrial genomes (Table 1), but most of this sequence (>650 kb) is only shared between *S. noctiflora* and *S. conica* and not with any other angiosperms. Overall, >85% of the voluminous intergenic sequence in these two species lacks detectable homology with any of the nuclear, plastid, or mitochondrial sequences available in the GenBank nr/nt database.

**Figure 5:**
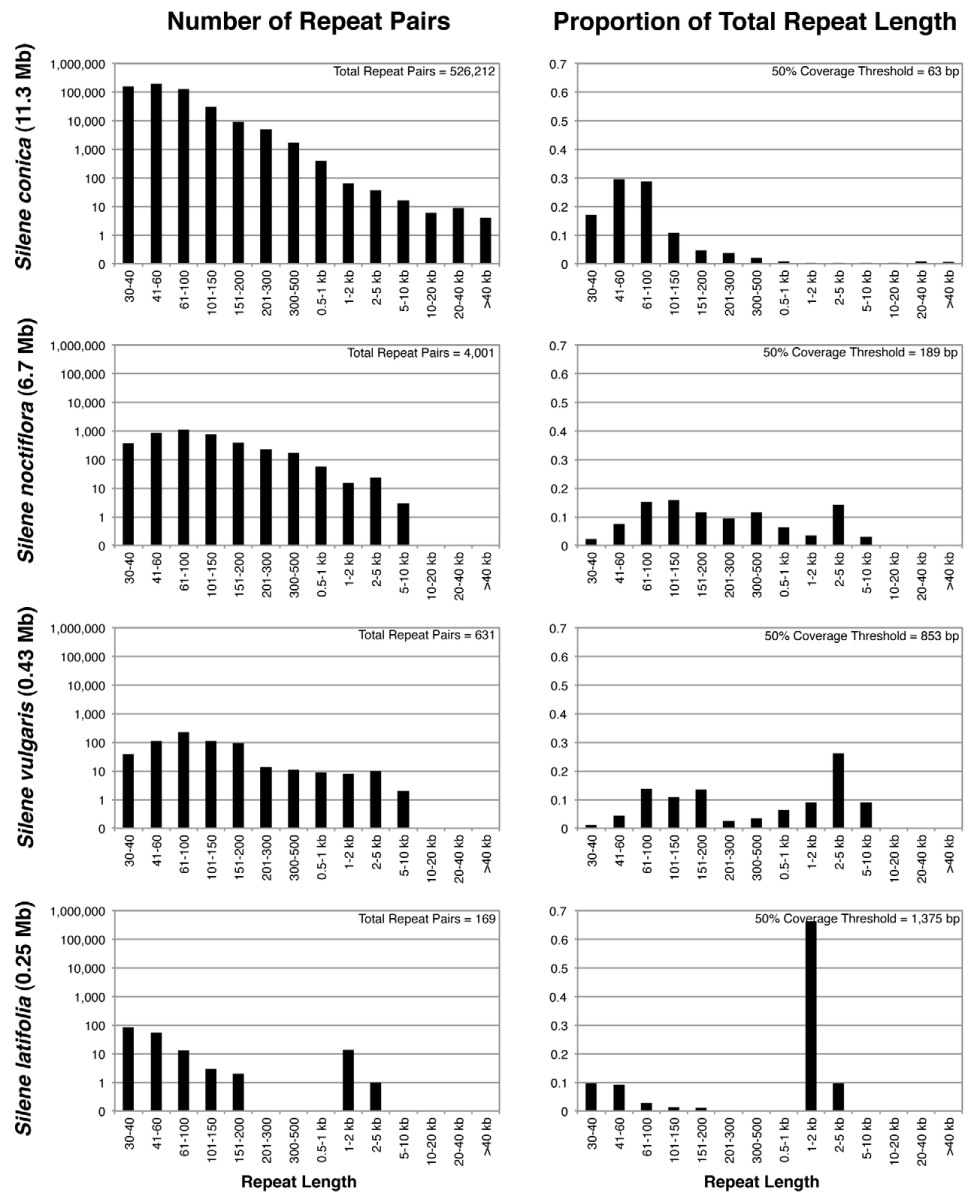
Size distribution of repetitive content by the number of repeat pairs (left column) and total repeat length (right column). Both datasets are based on all repeat pairs identified with BLAST by searching each genome against itself. Note that this method is different than counting individual repeat copies, which cannot be unambiguously identified when repeats exist in numerous partially overlapping copies, as they do in these genomes. For example, a repeat with four *copies* would be associated with six unique repeat *pairs*. Because of the enormous number of multicopy, overlapping repeats in *S. conica*, the total length of repeat pairs exceeds the size of the genome even though more than half of it is single-copy. For these same reasons, the distribution of repeat lengths in this figure differs from the repeat coverage statistics reported in Table 1, which consider what fraction of the genome is covered by repeats but not the total number of repeat pairs. The reported 50% coverage threshold represents the median of the total repeat length distribution.

Repeated sequences constitute a variable and often large component of seed plant mitochondrial genomes [40], and *Silene* species are noteworthy in both respects (Figures 5, S2, and S3; Table 1). The *S. conica* mitochondrial genome contains a remarkable 4.6 Mb of dispersed repeats, which is more than any other sequenced plant mitochondrial genome in both absolute and percentage (40.8%) terms [40]. The largest repeats are >80 kb in size, but the bulk of the repetitive content consists of an enormous number of small, imperfect, and often partially overlapping repeats (Figures 5, S2, and S3). In contrast, repeat sequences make up just 6.7%–18.8% of the other three *Silene* mitochondrial genomes.

### Multichromosomal Mitochondrial Genome Structures

*Silene noctiflora* and *S. conica* have also evolved extraordinary mitochondrial genome structures. Although the relationship between genome maps and in vivo physical structure remains uncertain for angiosperm mtDNAs [41], the entire sequence content of the genome typically can be mapped as a single “master circle,” which can be subdivided into a collection of “subgenomic circles” that arise via high-frequency recombination between large direct repeats (Figure S4A) [42],[43]. This model applies to *S. latifolia* [38], whereas the *S. vulgaris* genome assembles into four circular-mapping chromosomes, with the largest (394 kb) comprising most (92%) of the genome and containing numerous repeats inferred to undergo active recombination on the basis of their association with alternative rearranged genome conformations (Figure S4). Two of the three smaller mitochondrial chromosomes in *S. vulgaris* share recombinationally active repeats with the large chromosome, but the majority of sequencing reads support the smaller subgenomic conformations (see Materials and Methods and Figure S4). In contrast, the smallest of the four *S. vulgaris* chromosomes appears to be almost completely autonomous. It does not share any repeats longer than 100 bp with the rest of the genome, and in the case of all shorter repeats shared between the smallest chromosome and the main chromosome, >99.5% of sequencing read-pairs support the smaller subgenomic conformation. While the presence of this small chromosome is itself unusual for plant mtDNAs, far more extreme are the *S. noctiflora* and *S. conica* mitochondrial genomes, each of which assembled into dozens of mostly autonomous and relatively small, circular-mapping chromosomes. The *S. noctiflora* mitochondrial genome consists of 59 circular-mapping chromosomes ranging from 66 to 192 kb in size (Table S2). Many of these do not share any large (>1 kb) repeats with other chromosomes. Even when *S. noctiflora* chromosomes do share large repeats (up to 6.3 kb), the clear majority of paired-end sequencing reads (>90% in all cases) support the conformation consisting of two smaller circles rather than a single combined circle. Although the extremely repetitive nature of the *S. conica* mitochondrial genome precluded complete genome assembly, its structural organization is similar to that of *S. noctiflora*. The vast majority (98.2%) of sequence content assembled into 128 circular-mapping chromosomes ranging from 44 to 163 kb in size (Table S2). Most of these chromosomes share only short repeats with other parts of the genome.

The number of sequencing reads that cover a given position in a shotgun genome assembly (i.e., the read depth) can be used to estimate the relative abundance of different sequences. The difference in average read depth between the chromosomes with the highest and lowest coverage was only 1.7-fold in *S. noctiflora* and only 3.1-fold in *S. conica* (after excluding repetitive regions), indicating that the abundance of the numerous chromosomes was relatively even in both genomes. The different chromosomes also exhibited a high degree of similarity in GC content within each genome (Table S2).

Assembly of repetitive genomes is inherently complicated, and this is particularly relevant to the identification of genomic subcircles because tandem duplications within a larger chromosome can misassemble as subcircles. However, such assembly errors leave clear signatures, including dramatic variation in read depth and conflicting read-pairs associated with the boundary between tandem repeats and flanking regions. The absence of such patterns in our dataset indicates that the assembled circles are not an artifact of tandem repeats within larger chromosomes. Nevertheless, it is possible, particularly in the draft assembly of *S. conica* mitochondrial genome, that some repeat pairs have been “collapsed” into single sequences, leaving open the possibility that the reported 11.3 Mb genome size for *S. conica* is a slight underestimate.

### Repeat-Mediated Recombinational Activity

**Figure 6:**
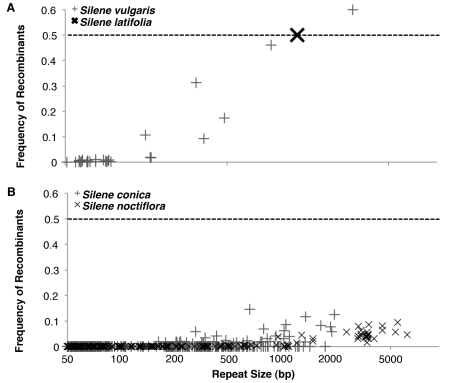
Repeat-mediated recombinational activity in the low mutation rate *S. latifolia* and *S. vulgaris* mitochondrial genomes (A) and the fast-evolving *S. noctiflora* and *S. conica* mitochondrial genomes (B). Each point represents a pair of repeats, and its position on the *y*-axis denotes the proportion of recombinant genome conformations detected with paired-end 454 reads. The dashed lines indicate the level at which equal frequencies of read pairs support recombinant and nonrecombinant conformations. The *S. latifolia* mitochondrial genome was not sequenced with 454 paired-end reads, but Southern blot hybridizations indicated that alternative genome conformations associated with its six-copy 1.4-kb repeat exist at roughly equivalent frequencies [38], as indicated by the large X.

Sequencing of the *S. latifolia* mitochondrial genome showed that it contains a six-copy 1.4-kb repeat that is highly recombinationally active with physical cross-overs between repeat copies generating a suite of rearranged genome conformations [38]. Southern blot analysis confirmed that the many alternative genome conformations occur in roughly equivalent frequencies in *S. latifolia* [38]. Paired-end sequencing reads can also be used to quantify the relative abundance of alternative genome conformations (see Materials and Methods and Figure S4), and our 454 data suggest a comparably high level of repeat-mediated recombinational activity for the largest repeats in the *S. vulgaris* mitochondrial genome (Figure 6A). The relative frequency of recombinant genome conformations increases with repeat size, and all surveyed repeats longer than 100 bp exhibit evidence of a history of recombination. The two largest surveyed pairs of repeated sequences (0.9 and 3.0 kb) in the *S. vulgaris* genome each appear to be at or near a 50∶50 level of alternative genome conformations (Figure 6A).

**Figure 7:**
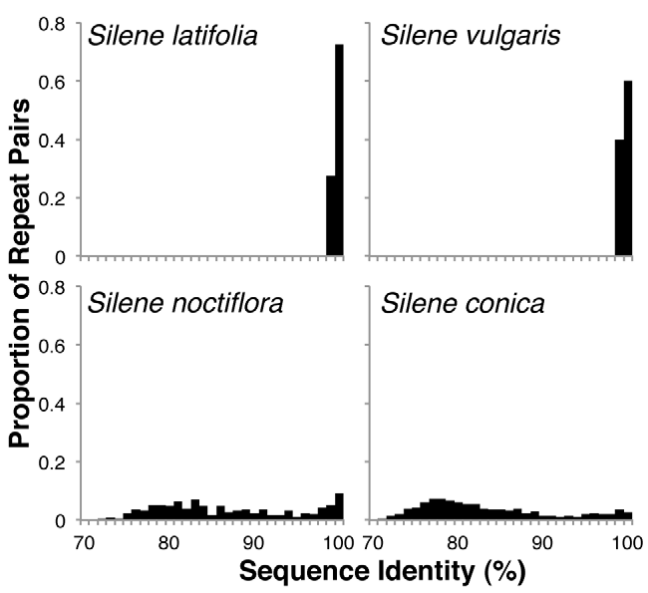
Distribution of percent sequence identity between pairs of repeats detected by BLAST. Only repeat pairs greater than 300 bp in length were used to calculate these distributions.

The rapidly evolving mitochondrial genomes of *S. noctiflora* and *S. conica* exhibit reduced frequencies of recombinant genome conformations compared to other *Silene* genomes (Figure 6B) and all other angiosperm mitochondrial repeats for which recombinational activity has been assessed. Even the largest repeats in the *S. noctiflora* genome (up to 6.3 kb) are associated with only a small minority of recombinant products (Figure 6B). The largest repeats in the *S. conica* genome (up to 87 kb) far exceed our paired-end library span and therefore cannot be analyzed for recombinational activity, but analysis of the shorter repeats suggests that the genome has experienced a similar shift in the relationship between repeat length and the frequency of recombinant products (Figure 6B). Recombinational activity (including gene conversion) is expected to homogenize copies of repeated sequences throughout the genome. Therefore, the dramatic increase in the proportion of divergent pairs of repeated sequences within the mitochondrial genomes of *S. noctiflora* and *S. conica* (Figures 7 and S5) is consistent with a reduction in recombinational activity in these species, though the existence of divergent repeats could also result from the increased mutation rate in these species or a reduced probability of gene conversion events between physically disparate repeat copies in expanded genomes.

### Maternal Inheritance of *Silene* Mitochondrial Genomes

The coexistence of maternally and paternally derived mitochondrial genomes in a heteroplasmic state within the same individual or maternal family would introduce complications for genome sequencing and assembly. Therefore, we looked for evidence of heteroplasmy and nonmaternal inheritance in the families used in this study. *S. vulgaris* has been the subject of extensive investigation into the patterns of mitochondrial genome inheritance [44]–[47]. These studies have found that mtDNA transmission is predominantly maternal in *S. vulgaris*, with a low frequency of biparental inheritance or paternal “leakage.” Because of this evidence, the *S. vulgaris* family used for genome sequencing was chosen, in part, because the maternal source plant had previously been screened with two highly polymorphic mitochondrial markers and revealed no evidence of heteroplasmy [46]. Although similarly intensive investigations of mtDNA inheritance have not been performed in other *Silene* species, we found evidence of maternal transmission in *S. latifolia*, *S. noctiflora*, and *S. conica*. An analysis of cleaved amplified polymorphic sequences (CAPS) showed that all progeny (16–48 per species) from controlled greenhouse crosses inherited the maternal variant of a SNP. Mitochondrial inheritance therefore appears to be at least predominantly maternal in all four *Silene* species, making it unlikely that genome assembly complications arising from biparental inheritance and heteroplasmy can explain the observed differences in mitochondrial genome size and complexity among *Silene* species.

### Intraspecific Nucleotide Polymorphism

*S. noctiflora* and *S. conica* do not show the proportional increases in mitochondrial nucleotide diversity that would be expected on the basis of their accelerated mutation rates (even after accounting for the approximately 2-fold differences in generation times across the four *Silene* species [48]), suggesting a recent history of lower effective population size (*N*_e_) than their congeners and/or a recent reversion to lower mitochondrial mutation rates as observed in other accelerated angiosperm lineages [33],[34]. In *S. conica*, there is less than a 10-fold increase in mitochondrial synonymous nucleotide diversity relative to the more slowly evolving *Silene* species, and *S. noctiflora* exhibits no sequence variation whatsoever across our sample of mitochondrial, plastid, and nuclear loci (Table S3) (see also [49]).

## Discussion

### The Mysterious Origins of Expanded Intergenic Regions in Plant mtDNA

**Figure 8:**
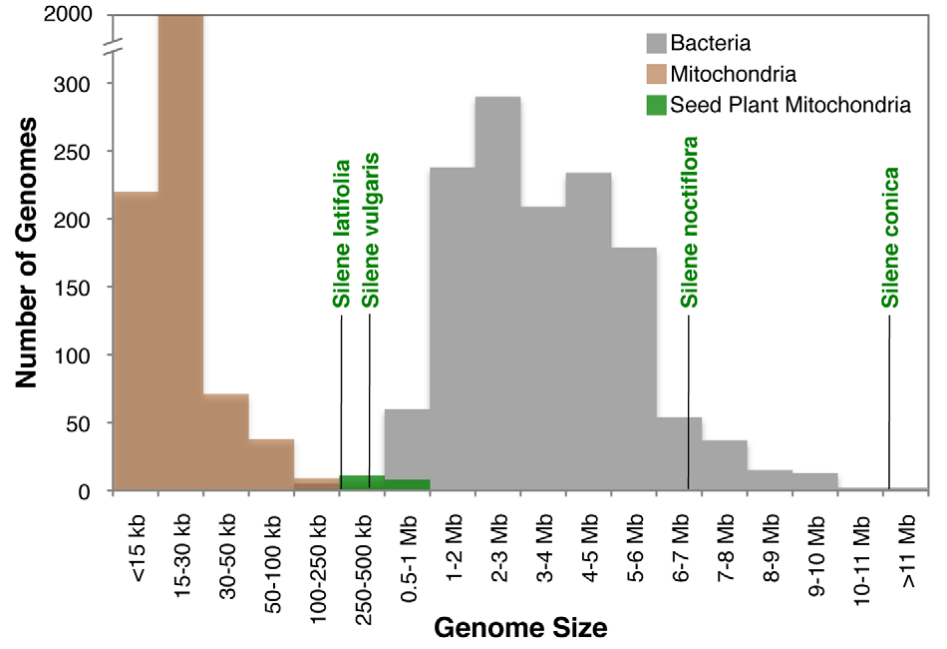
*Silene* mitochondrial genome sizes relative to all sequenced mitochondrial and eubacterial genomes from the National Center for Biotechnology Information (NCBI) Genome database.

The dramatic expansion of intergenic content in the mtDNA of *S. noctiflora* and *S. conica* has resulted in mitochondrial genomes that are larger than most bacterial genomes (Figure 8) and even some nuclear genomes [50]. These enormous genomes add to the long-standing mystery regarding the origins of intergenic sequences in plant mtDNA [28].

It is possible that a significant portion of this intergenic content is derived from the nuclear genome, for which sequence data are still limited in *Silene*. However, by comparing the mitochondrial genomes against a large set of cDNA sequences derived from a recent transcriptome project in *S. vulgaris* [51], we detected similarity for only a trivial amount (<0.1%) of the otherwise uncharacterized mitochondrial sequence in *S. noctiflora* and *S. conica*. Therefore, if nuclear DNA is a major contributor to the expanded mitochondrial intergenic regions in these species, it is most likely drawn from the vast repetitive and noncoding fractions of the nuclear genome. That the origin of only a small fraction of the intergenic sequences in *S. noctiflora* and *S. conica* can be identified may reflect the rapid rates of sequence and structural divergence in these mitochondrial genomes.

In other plant mitochondrial genomes, the proliferation of “selfish” DNA may have contributed to expansions in intergenic regions. For example, the mtDNA of the gymnosperm *Cycas* contains numerous copies of repetitive elements known as Bpu sequences [52], and the expanded mitochondrial and plastid genomes of the green alga *Volvox* share an apparently self-replicating element with the nucleus [53]. The finding of expanded intergenic sequence in *S. noctiflora* and *S. conica* mtDNA raises the question of whether some form of selfish element has been involved. This appears possible in *S.* conica, given the highly repetitive nature of its mitochondrial genome (Figures 5, S2, and S3; Table 1). However, we did not find evidence for any specific sequence or set of sequences that dominate the repetitive content in *S. conica*. There is even less evidence for a role of mobile, self-replicating elements in *S. noctiflora* mtDNA given the small amount of repeated sequence in this genome. Interestingly, *S. noctiflora* harbors a relatively modest proportion of repetitive sequence compared to many other angiosperms' mtDNAs, including the much smaller *S. vulgaris* genome (Figures 5 and S2; Table 1), indicating that there is no strict relationship between repetitive content and genome size.

It is noteworthy that *S. noctiflora* and *S. conica* share a large amount of intergenic sequences with each other (659 kb and 760 kb, respectively) that show little or no homology with any available sequences in the GenBank nr/nt database including all other sequenced plant mitochondrial genomes. These shared intergenic sequences may be the remnants of an ancestral genomic expansion that preceded the divergence of *S. noctiflora* and *S. conica*, suggesting a possible sister relationship between these two lineages, an issue that is currently unresolved by molecular phylogeny [37],[54]. If so, this could indicate that the atypical mitochondrial genome size, structure, and substitution rates in *S. noctiflora* and *S. conica* represent a single set of evolutionary changes rather than phylogenetically independent events. However, we cannot rule out the possibility that the shared sequences are the result of parallel acquisitions from similar sources, such as the nuclear genomes in each species. Generating sequence data from other genomic compartments, particularly from a large number of unlinked nuclear loci, should provide better insight into the phylogenetic history of these *Silene* species.

### The Evolution of Multichromosomal Mitochondrial Genome Structure

Although the highly multichromosomal genome structures observed in *S. noctiflora* and *S. conica* are novel for plant mitochondria, various forms of multicircular organelle genomes have evolved independently in diverse eukaryotic lineages, including in the mitochondria of kinetoplastids [55], diplonemids [56], chytrid fungi [57], and a number of atypical metazoans [58]–[61], as well as in dinoflagellate plastids [62]. In addition, the recent analysis of the cucumber mitochondrial genome showed that a small fraction of that genome can be mapped to two circular chromosomes that appear to be independent from the main chromosome [63].

It should be noted that the maps generated from the assembly of DNA sequence data do not necessarily reflect the structure of the genome in vivo. In particular, linear concatamers and overlapping linear fragments can assemble as circular maps [64]. Efforts to directly observe the molecular structure of angiosperm mitochondrial genomes have identified a complex mixture of linear, circular, and branched molecules [65],[66], indicating that the circular maps produced by genome projects may be abstractions or oversimplifications. Although on the basis of our current data we cannot distinguish between the various structural alternatives capable of producing circular chromosome maps, the sequence assemblies do support the intriguing finding that many of these chromosomes are structurally autonomous, lacking the large, recombinationally active repeats that are characteristic of most angiosperm mitochondrial genomes.

The existence of multichromosomal mitochondrial genomes in *Silene* raises fundamental questions about the nature of replication and inheritance of these genomes. Notably, we did not detect a single intact gene in many chromosomes, including the smallest chromosome in *S. vulgaris*, 20 of the 59 chromosomes in *S. noctiflora*, and 86 of the 128 chromosomes in *S. conica* (note that these totals do not include chromosomes in *S. noctiflora* and *S. conica* that only contain partial gene fragments that require *trans*-splicing with transcripts originating from other chromosomes to generate complete coding sequences). Therefore, the functional significance (if any) of these “empty” chromosomes and the evolutionary forces that maintain their presence and abundance within the mitochondrion are unclear. While it is possible that these chromosomes contain unidentified genes or noncoding elements that are functionally important and therefore conserved by selection, they may also replicate and proliferate in a nonadaptive or even selfish fashion.

Our analysis was based on mtDNA extracted from predominantly vegetative tissue pooled across multiple individuals from a single maternal family. Therefore, we do not know whether any of the observed structural variation in mtDNA is partitioned within our pooled sample and, if so, at what level it is partitioned (i.e., among individuals, tissue types, cells, or even individual mitochondria). In this light, it would be particularly informative to conduct an analysis of mitochondrial genome sequence and structure in meristematic tissue to compare with our results from vegetative tissue. Any differences between these tissue types would be of interest because the mtDNA in meristematic tissue should better represent the inherited form of the genome.

### The Mutational Burden Hypothesis and the Evolution of Genome Architecture

The co-occurrence of mutational acceleration and genome expansion in the mitochondria of *S. noctiflora* and *S. conica* runs counter to patterns in other eukaryotic mitochondrial genomes (e.g., plants versus animals). Although we cannot determine the relative timing of these changes, their co-occurrence in these lineages is at odds with the hypothesis that reduced mutation rates are a major cause of mitochondrial genome expansion in plants [32].

An alternative possibility that would be consistent with the MBH is that these species have a small *N*_e_, which has reduced the efficacy of selection against the proliferation of noncoding elements even if the intensity of that selection has increased with higher mutation rates. There is some evidence to support this possibility, particularly in *S. noctiflora*, which appears to have a very low *N*_e_ based on the striking lack of polymorphism in genes from all three genomes (Table S3) [49]. However, the finding of high levels of mitochondrial polymorphism in *S. conica* (Table S3) is contrary to the predictions of the MBH. Some caution is warranted in interpreting the nucleotide diversity data because standing levels of polymorphism are very sensitive to recent bottlenecks and do not necessarily represent the long-term average *N*_e_ over the entire history of a species or lineage. One alternative proxy for *N*_e_ and the relative strength of genetic drift is the ratio of nonsynonymous to synonymous substitutions (*d*_N_/*d*_S_), with higher ratios indicating a reduced efficacy of selection in purging deleterious changes in amino acid sequence [12]. Based on this alternative measure, there is no indication of a long-term decrease in *N*_e_ in either *S. noctiflora* or *S. conica* since their divergence from the other *Silene* species (Table 1). Therefore, with respect to both mutation rate and *N*_e_, the changes in mitochondrial genome size within *Silene* appear to be inconsistent with any straightforward interpretation of the MBH.

In contrast to the differences in overall genome size in *Silene* mitochondria, some of the observed changes in these genomes are consistent with predictions of the MBH. Most notably, average intron lengths have decreased in the species with elevated mutation rates, and the only example of an intron loss was observed in a high-rate species. These results could indicate that the consequences of mutational burden vary substantially within a genome. For example, the contrasting patterns observed in introns versus intergenic regions within these lineages might suggest that the burden associated with disruptive mutations in functional noncoding elements such as introns is of far greater evolutionary importance than that associated with gain-of-function mutations creating novel deleterious elements in largely nonfunctional intergenic regions.

The inability of existing theory to fully account for the extreme patterns of divergence in *Silene* mitochondrial genomes points to a valuable opportunity to expand our understanding of the evolutionary forces that shape genomic complexity. Although this study was restricted to a small number of species from a single genus, it captured enormous variation in genome architecture (e.g., approximately 98% of the known range of organelle genome sizes), indicating that profound and perhaps novel evolutionary mechanisms are acting to shape mitochondrial genome size and complexity in *Silene*.

### Recombination, Gene Conversion, and the Maintenance of Plant Mitochondrial Genome Stability

The observed differences among *Silene* species in the frequency of recombinant genome conformations raise the possibility that recombination could be a key factor underlying the extreme patterns of mitochondrial genome evolution in *S. noctiflora* and *S. conica*. The mitochondrial genomes in these species differ from those of other angiosperms in numerous respects, including rates of point mutations and indels, presence of duplicated and divergent gene copies, frequency of RNA editing, genome size, and structural organization (Table 1). Many, perhaps all, of these traits are likely affected by the related processes of intragenomic recombination and gene conversion.

Recombinational processes play an important role in plant mitochondrial genome sequence and structural evolution [43],[67]. In addition, recombination between repeated sequences (including very short repeats) has been shown to be an important mechanism for sequence deletion in plant nuclear genomes [68]. Therefore, changes in recombinational activity are expected to affect the evolution of genome size. However, recombinational processes can also have opposing effects on genome size via sequence duplication or integration of new content, so that the relationship between recombination and genome size is likely to be a complex one. Recombination and gene conversion mechanisms have also been implicated in the evolution of other elements of genome architecture. For example, retroprocessing events involving cDNA intermediates are likely responsible for the loss of introns and RNA editing sites [34],[69],[70].

Recombination and gene conversion are key components of DNA repair pathways. Notably, gene conversion mechanisms that are biased against new mutations have been proposed to slow the effective or observed mutation rate in multicopy genomes [71],[72]. Our findings raise the possibility that template-based recombinational repair and biased gene conversion are important factors underlying the typically low rates of nucleotide substitution in plant mitochondrial genomes and that these mechanisms have been altered or disrupted in fast-evolving species such as *S. noctiflora* and *S. conica*. The associated increase in the rate of mitochondrial indels in these species (Figure 3) suggests that alterations in replication and repair machinery can have correlated effects on both point mutations and structural changes, which is consistent with the correlation between rates of mitochondrial sequence and structural evolution observed in other lineages [73]–[76].

Our findings highlight the need to characterize *Silene* nuclear gene families involved in recombination and other aspects of organelle genome maintenance. Unraveling the process of sequence gain and turnover in these rapidly evolving mitochondrial genomes should provide insight into the evolutionary forces underlying the tremendous variation in size and complexity of eukaryotic genomes.

## Materials and Methods

### Study System

The genus *Silene* (Caryophyllaceae) consists of approximately 700 predominantly herbaceous species of flowering plants [77], many of which are used as models in ecology and evolution [78]. *S. noctiflora* L. and *S. conica* L. both have annual life histories [79], and they are largely hermaphroditic but produce a low frequency of pistillate (female) flowers and can therefore be characterized as gynomonoecious [80]–[82] (DBS, personal observation). *S. latifolia* Poir. and *S. vulgaris* (Moench) Garcke are short-lived perennials with an average generation time of approximately 2 y [48] that maintain dioecious and gynodioecious breeding systems, respectively [79],[80].

### Source Material and mtDNA Extraction

Details of the *Silene latifolia* mitochondrial genome project were described previously [38]. For each of the other three species, approximately 200 g of tissue was collected from multiple individuals of a single maternal family. The maternal lineages were derived from seeds originally collected in Abruzzo, Italy (*S. conica*), Eggleston, VA, US (*S. noctiflora*), or Stuarts Draft, VA, US (*S. vulgaris*). Voucher specimens from each of these maternal lineages have been deposited to Massey Herbarium at Virginia Polytechnic and State University: *S. conica* (L Bergner 003), *S. noctiflora* (D Sloan 003), *S. vulgaris* (L Bergner 007).

All aboveground tissue was used for *S. vulgaris*, including leaves, stems, and flowers, while only leaf tissue was collected for *S. noctiflora* and *S. conica*. Mitochondrial DNA was purified from mitochondria from harvested tissue using established protocols based on differential centrifugation, treatment with DNase I, and then either CsCl gradients or phenol∶chloroform extraction [83],[84]. Restriction digests with MspI and HpaII enzymes, which share identical recognition sequences but differ in methylation sensitivity, were performed to confirm the absence of significant nuclear contamination from the purified mtDNA samples prior to sequencing.

### 454 and Illumina Sequencing

For each of the species, 3-kb paired-end libraries were prepared following standard protocols for sequencing on a Roche 454 GS-FLX platform with Titanium reagents. Additional libraries were prepared (also following standard Roche protocols) for the larger *S. noctiflora* and *S. conica* mitochondrial genomes, including shotgun libraries for both species and a 12-kb paired-end library for *S. noctiflora*. The latter was constructed following the standard 8-kb protocol, but the larger 12-kb average fragment size range was selected on the basis of the size distribution of the DNA sample after shearing. Each library was run on a single quarter-plate region except for the *S. conica* shotgun library and the *S. noctiflora* 12-kb paired-end library, which were each run on two quarter-plate regions. The shotgun library for *S. noctiflora* was constructed and sequenced by the Genome Center at Washington University in St. Louis (MO, US). All other 454 library construction and sequencing was performed at the Genomics Core Facility in the University of Virginia's Department of Biology.

To generate sufficient starting material for Illumina library construction, mtDNA samples were amplified with GenomiPhi V2 (GE Healthcare). Paired-end sequencing libraries were generated and tagged with multiplex barcodes using the NEBNext DNA Sample Prep Reagent set 1 (New England Biolabs) in accordance with protocols developed by the University of California Davis Genome Center. In brief, DNA samples were sonicated to a peak fragment size of between 300 and 600 bp. DNA fragments were then end polished and ligated to adaptors carrying a unique 6-bp barcode. The resulting samples were gel-purified and amplified with 14 PCR cycles using paired-end library primers. The three libraries were included in a larger sample pool and sequenced in a single lane of a 2×85 bp paired-end run on an Illumina GAII. Sequencing was performed at the Biomolecular Research Facility in the University of Virginia's School of Medicine.

### Genome Assembly

Each quarter-plate 454 run produced between 32 and 104 Mb of sequence. The total sequencing yield was 270, 210, and 51 Mb for the *S. noctiflora*, *S. conica*, and *S. vulgaris* mtDNA samples, respectively. However, not all sequence data were used in primary genome assembly. For *S. noctiflora*, only the shotgun and 3-kb paired-end data were analyzed in the initial assembly process. The 12-kb paired-end data were only used to resolve structures associated with large (>3 kb) repeats and to quantify the frequency of alternative genome conformations resulting from recombination among repeat copies (see below). For the smaller, *S. vulgaris* mitochondrial genome, a single quarter-plate run produced very high coverage (>80×). Preliminary analyses suggested use of the entire dataset increased fragmentation in the assembly. Therefore, a random set of sequence reads totaling 25 Mb was selected for initial assembly. The full *S. vulgaris* dataset was used for subsequent quantification of alternative genome conformations.

For each genome, the 454 sequence reads were assembled with Roche's GS de novo Assembler v2.3 (“Newbler”) using default settings. The resulting assemblies produced average read depths of 20×, 25×, and 42× for the *S. conica*, *S. noctiflora*, and *S. vulgaris* mitochondrial genomes, respectively. Although the assemblies contained few, if any, gaps or low-coverage regions, they were highly fragmented because of the repetitive and recombinational nature of these genomes (Figures 5 and 6). The assemblies also contained contigs from contaminating nuclear, plastid, and viral DNA. True mitochondrial contigs were distinguished on the basis of read depth and connectivity to other contigs in the assembly, which was inferred from two types of data: (1) paired-end reads that mapped to two different contigs and (2) single reads that were split by the assembler and assigned to the ends of two different contigs. On the basis of these data, contigs were organized into “subgenomes,” each of which represented either a closed circular assembly or a single-copy assembly flanked on either side by recombinationally active repeats. Each of these subgenomic contig groups was then reassembled using a custom set of Perl and BASH scripts that identified all sequencing reads uniquely associated with the corresponding contigs and ran a new assembly using only those reads. The resulting subgenomic assemblies were then manually edited and combined as necessary with the aid of Consed v17.0 [85].

The largest repeats in both the *S. conica* and *S. vulgaris* mitochondrial genomes exceed the 3-kb span size of their respective paired-end libraries. Therefore, the relationships between the single-copy regions flanking these large repeats are ambiguous. These ambiguities were tentatively resolved on the basis of the pattern observed in smaller repeats within each genome (Figure 6).

On the basis of the high level of recombinational activity among smaller repeats in *S. vulgaris*, we assumed that large repeats also have high recombinational activity. Therefore, we assembled the majority of the *S. vulgaris* genome content into a single chromosome, analogous to the “master circle” typically reported for plant mitochondrial genomes. This large chromosome contains numerous recombinationally active repeats, and, as discussed previously [38], the arrangement of repeats and single-copy regions within this chromosome should be considered only one of many possible alternative representations. We also identified three small circular-mapping structures that were not included in the main assembly. One of these circles (Chromosome 4) shows almost no evidence of recombinational activity with the rest of the genome, while the other two do share repeats that appear to recombine frequently with the main chromosome. However, in both of these cases, the repeats are small (<500 bp), and the clear majority of reads support the closed circle conformations over a single combined circle. For convenience, we refer to these three circles as chromosomes, but their small size and (in the case of Chromosomes 2 and 3) substantial degree of recombinational activity with the rest of the genome distinguish them from the chromosomal structure that characterizes the *S. noctiflora* and *S. conica* mitochondrial genomes.

In contrast to *S. vulgaris*, the bulk of the *S. noctiflora* and *S. conica* mitochondrial genomes map to discrete circular chromosomes that exhibit little or no recombinational activity with the rest of the genome. In both species, repeats show much less evidence of recombination than repeats of similar size in *S. latifolia* and *S. vulgaris* (Figure 6). Moreover, in cases of recombinationally active repeats, the clear majority of paired-end reads (>90% in all cases in *S. noctiflora* and the vast majority of cases in *S. conica*; Figure 6) support minimally sized circular conformations rather than larger combined circles. Therefore, for assembly ambiguities associated with repeats exceeding the 3-kb paired-end library span in *S. conica*, it was assumed that minimally sized circles predominate over larger combined conformations.

### Mapping Illumina Sequence Data

To correct base-calling errors including insertion and deletion errors known to be associated with long single-nucleotide repeats (i.e., homopolymers) in 454 sequence data, we mapped Illumina sequence data onto the completed mitochondrial genome assemblies for each species. After removal of multiplex barcodes and quality trimming, Illumina sequencing yielded average read lengths between 53 and 69 bp with a total of 398, 326, and 168 Mb of sequence data for *S. noctiflora*, *S. conica*, and *S. vulgaris*, respectively. Paired-end read mapping was performed with SOAP v2.20 [86] with the following parameters: m 100, x 900, g 3, r 2. A set of custom Perl scripts were used to call SOAP, parse the resulting output, and modify the genome sequence on the basis of well-supported sequence conflicts. These scripts were run recursively until additional iterations did not produce any further improvement to the sequence.

For both *S. vulgaris* and *S. noctiflora*, Illumina mapping provided high-depth (>10×) coverage for essentially the entire genome (>99.9%). This process identified 55 sequence corrections in *S. vulgaris* and 1,734 corrections in *S. noctiflora*, the vast majority of which were associated with homopolymer runs. In contrast, because of the larger size and repetitive complexity of the *S. conica* mitochondrial genome, more than 10% of the sequence had coverage levels below 10×. Furthermore, the recursive mapping approach described above failed to converge for numerous regions in the genome, indicating low confidence in many of the sequence corrections indicated by the Illumina data. To avoid incorporating false sequence changes, we did not use the Illumina data to perform genome-wide corrections in *S. conica*. Consequently, the reported genome sequence likely contains some errors associated with homopolymer runs. We did, however, use the Illumina data to verify basecalls in *S. conica* coding genes and introns, including cases of frameshift mutations.

### Gene Annotation and Characterization of Intergenic Content

The annotation of protein, rRNA, and tRNA genes was performed using a combination of local BLAST [87] and tRNAscan [88] as described previously [20]. Annotated genome sequences were deposited in GenBank (Table S2).

To identify sequence of plastid origin in the *Silene* mitochondrial genomes, each genome was searched against a database of seed plant plastid genomes, using NCBI-BLASTN (v2.2.24+) with the following parameter settings: dust no, gapopen 8, gapextend 6, penalty -4, reward 5, word_size 7. Only hits with a raw score of at least 250 were considered. These hits were subsequently filtered to exclude matches involving mitochondrial protein and rRNA genes known to have ancient plastid homologs (e.g., mitochondrial *atp1* and plastid *atpA* [89]). We also excluded hits with very high AT contents (>72%), because we found these to be almost exclusively false positives resulting from the use of sensitive BLAST parameters.

To identify intergenic sequence conserved in other plant mitochondrial genomes, all intergenic regions (excluding those of plastid origin) were searched against a database of all sequenced seed plant mitochondrial genomes using NCBI-BLASTN (v2.2.24+) and the following search parameters: task blastn, dust no, gapopen 5, gapextend 2, reward 2, penalty -3, word_size 9. All hits with a raw score of at least 70 were considered homologous. Note that we included all sequences from “empty” chromosomes in the intergenic category even though such sequences are not technically bounded by genes on either side.

To identify additional conserved sequences (particularly ones of nuclear origin), the remaining intergenic regions (i.e., excluding annotated genes, plastid-derived sequence, and regions conserved with other plant mitochondrial genomes) were searched against the GenBank nr and nt databases (release date 12/15/2010) using NCBI-BLASTX and BLASTN (v2.2.24+). Default settings were used for BLASTX, whereas the BLASTN search parameters were as follows: dust yes, gapopen 5, gapextend 2, reward 2, penalty -3, word_size 9. All BLASTX hits with a raw score of at least 140 and all BLASTN hits with a raw score of 70 or above were considered homologous. Searches with these same parameters were also conducted against a set of assembled cDNA sequences from a recent *S. vulgaris* transcriptome project [51].

### Characterization of Repetitive Content

Tandem repeats in each *Silene* mitochondrial genome were identified with Tandem Repeat Finder v4.04 [90], but these represented a negligible fraction of total repeat content in each genome and are not reported separately. Dispersed repeats were identified by searching each genome against itself with NCBI-BLASTN (v2.2.24+) using default parameter settings. All hits with a raw score of at least 30 were considered repeats. The shortest possible sequence that can satisfy this criterion is a perfect 30-bp repeat, but longer sequences with less than 100% sequence identity can also be identified by this method. Finally, Vmatch (http://www.vmatch.de) was used to precisely define the boundaries of all repeats with 100% sequence identity.

### Analysis of Recombinational Activity

We used paired-end reads from 454 sequencing to quantify the relative abundance of alternative genome conformations associated with repeat-mediated recombination (Figure S4). In the absence of any recombination or alternative genome conformations, 454 read pairs should map to positions in the genome that are consistent with the size span of the sequencing library (∼3 or 12 kb in this case). However, the presence of genomic rearrangements will result in read pairs that are inconsistent with the reported genome conformation (Figure S4). Therefore, for each pair of repeated sequences in a genome, we quantified the number of 454 read pairs that are inconsistent with the reported genome assembly but are consistent with either of the predicted products of recombination between the repeats. This number was then compared against the total number of consistent read pairs in the genome that span one of the two repeat copies to determine the relative abundance of the recombinant products.

To perform this analysis, 454 paired-end reads were mapped on the corresponding genome sequence using Roche's GS Reference Mapper v2.3 software with default parameters. For *S. noctiflora*, only reads from the 12-kb paired-end library were used. The resulting output was filtered to exclude duplicate read pairs with identical start positions for both the left and right sequences, as these were assumed to have been generated by the PCR amplification step in paired-end library construction, making them nonindependent data points. Inspection of the mapping output suggested that the analysis was too stringent in identifying consistent read pairs. Therefore, any “inconsistent” read pairs that mapped in a proper orientation within a distance of 4–16 kb for a 12-kb library or 1–6 kb for a 3-kb library were reclassified as consistent. These size ranges were determined on the basis of manual inspection of the distribution of mapping spans.

Identified repeats within each genome (see above) were filtered on the basis of multiple criteria prior to inclusion in recombination analyses. First, only repeats of at least 50 bp in length and at least 95% sequence identity were considered. Additional repeat pairs were excluded because their proximity to each other or to other repeats would have led to ambiguity in the interpretation of paired-end mapping results. Specifically, repeats were excluded if the two copies were separated by less than the maximum library span or if there was a “correlated” pair of larger repeats within the maximum library span of each repeat copy. Finally, for *S. conica* and *S. vulgaris* (for which only 3-kb paired-end libraries were available), repeat pairs were excluded if one of the repeat copies was within 100 bp of the start of any other repeat >500 bp in size. These cases were excluded because the presence of adjoining repeats would preclude unambiguous mapping of reads to the flanking sequence. Because of the limited physical coverage and short (3 kb) span length in the *S. conica* paired-end data, there are many repeat pairs (particularly large repeats) in this genome that passed the aforementioned criteria, but have an insufficient number of read pairs to precisely measure the relative frequency of alternative genome conformations. Therefore, frequencies are only reported for repeat pairs that have at least five consistent read pairs spanning each copy. Finally, because of the enormous number of small repeats in the *S. conica* mitochondrial genome (Figure 5), only a random sample of 5% of repeat pairs shorter than 200 bp was included.

To validate our methodological approach, we ran a set of control analyses that used the same set of repeats except that we reversed the coordinates for one of the copies. Therefore, these analyses assessed rearrangements associated with the same genomic regions but would only detect alternative genome conformations if recombination occurred between two homologous sequences lined up in opposite orientations. The frequency of alternative genome conformations was at or near zero for every one of these control analyses (Figure S6). This suggests that baseline level of genome rearrangement and chimeric artifacts is very low in our dataset and that the alternate genome conformations detected by these methods are the genuine result of repeat-mediated recombination. In addition, the differences in assembly methods across species (see above) should have no effect on the reported estimates of recombinational activity because these differences only pertain to large repeats exceeding the span of our paired-end libraries, which were not assayed for recombination.

### Estimates of Nucleotide Substitution Rate

Previous analyses based on individual genes have identified massive variation in mitochondrial substitution rates among genes and species within the genus *Silene* [35]–[37],[91]. To assess these patterns at a genome-wide scale, all protein genes were aligned with MUSCLE v3.7 [92] and levels of synonymous (*d*_S_) and nonsynonymous (*d*_N_) divergence were estimated using PAML v4.4 [93] as described previously [37]. Analyses were run both on individual genes and on a concatenated dataset of all shared protein genes. Most analyses included six species (*Arabidopsis thaliana*, *Beta vulgaris*, and all four *Silene* species), but a larger dataset of sequenced seed plant mitochondrial genomes was also analyzed. In all cases, the phylogenetic relationships among the four *Silene* species were left unresolved (i.e., as a four-way polytomy), reflecting the apparently rapid radiation of these four lineages [37],[54]. Because substitutions at RNA editing sites can artificially inflate estimates of *d*_N_ [94], we excluded all codons that were found to be edited based on genome-wide datasets from four species [70],[95],[96]. To estimate absolute rates of nucleotide substitution in these genomes, *d*_N_ and *d*_S_ values were divided by an approximate divergence time of 6 Myr [35],[37],[97]. However, these estimates should be considered only rough approximations because of the uncertainty in divergence time [37] and the potential bias associated with recent polymorphisms [98],[99].

### Indel Analysis

To determine the frequency and size distribution of indels, all protein genes (including *cis*-spliced introns) from the four *Silene* species and the outgroup *B. vulgaris* were aligned with MUSCLE v3.7 and adjusted manually. Unalignable regions at the 5′ and 3′ ends of genes were excluded. The resulting alignments were analyzed to identify all indels that were unique to a single species and did not overlap with any other indels.

### Prediction of RNA Editing Sites

A genome-wide analysis of C-to-U RNA editing sites by cDNA sequencing has been reported previously for *S. latifolia* and *S. noctiflora* [70]. To estimate the frequency of RNA editing in *S. vulgaris* and *S. conica*, protein gene sequences were analyzed with a predictive algorithm (PREP-mt) [100]. Control analyses using *Silene* sequences with known editing sites suggested that different stringency settings (C-values) are appropriate for species with different rates of sequence evolution. Specifically, the *S. conica* data were analyzed with C = 0.8 and the *S. vulgaris* data were analyzed with C = 0.7. PREP-mt does not identify synonymous editing sites, so the reported totals were increased by 10% to approximate the contribution of synonymous edits on the basis of observed rates in other *Silene* genomes [70]. All intact protein genes were included as well as the following putative pseudogenes: *rps13* (*S. latifolia*), *rps3* (*S. conica*, *S. latifolia*, and *S. noctiflora*), and *ccmFc* (*S. conica*). For genes with duplicates within the genome, only a single gene copy was included.

### Estimating Nucleotide Polymorphism

To estimate levels of sequence variation within each of the four *Silene* species in this study, we PCR amplified and Sanger sequenced a sample of five mitochondrial loci as well as a single plastid and nuclear locus for multiple, geographically dispersed populations. Sequencing methods, source populations, and polymorphism data for *S. vulgaris* and *S. latifolia* were reported previously [36],[91]. Source populations for *S. noctiflora* and *S. conica* are summarized in Table S4. A single individual was sampled from each population. Sequence data from each species were analyzed with DnaSP v5 [101] to calculate nucleotide diversity and the number of segregating sites for each locus. Maximum likelihood estimates of Watterson's *Θ* and corresponding 95% confidence intervals were calculated as described previously [91]. For the nuclear *X4*/*XY4* locus, a single haplotype was randomly selected from each individual for calculation of polymorphism data. Only X-linked copies were included for *S. latifolia* males. Haplotypes were inferred from diploid sequence data using the program PHASE v2.1 [102]. Novel sequences were deposited in GenBank (accessions JF722621–JF722652).

### Testing for Maternal Inheritance of mtDNA

We performed a set of greenhouse crosses to test for maternal transmission of mtDNA in *S. latifolia*, *S. noctiflora*, and *S. conica* (*S. vulgaris* was not included because it has already been the subject of numerous studies examining mitochondrial genome inheritance and heteroplasmy [44]–[47]). Each cross involved an individual from the maternal family used for mitochondrial genome sequencing and an individual from another family in that species known to differ in mtDNA haplotype. For each species, a single pair of reciprocal crosses was performed, and a SNP was used to design a CAPS marker capable of distinguishing the two parental genomes (Table S5) [103]. For each pair of crosses, 16 to 48 progeny were analyzed with the corresponding CAPS marker.

## Supporting Information

Figure S1 **Lengths of** ***cis*** **-spliced introns in** ***Silene*** **mitochondrial genomes.**
(PDF)

Figure S2 **The effect of sequence identity and repeat length thresholds on estimates of repetitive content in** ***Silene*** **mitochondrial genomes.** Perfect repeats were identified with Vmatch, and imperfect repeats were identified with BLAST (see Materials and Methods). (A) The relationship between the percent of the genome covered by repeats and the minimum percent sequence identity (based on repeats pairs of at least 100 bp in length). (B) The relationship between the percent of the genome covered and repeat length (based on perfect repeat pairs only).
(PDF)

Figure S3 **Repeat coverage depth in** ***Silene*** **mitochondrial genomes.** For each curve, the *y*-intercept indicates the proportion of the mitochondrial genome that is single-copy in that species. Other points along the curve indicate the cumulative genomic coverage up to a certain repeat depth. For example, the height of the curve at a value of 10 on the *x*-axis indicates the fraction of the genome represented by all nucleotide positions that match nine or fewer repeats elsewhere in the genome. The lower position of the *S. conica* curve reflects the highly repetitive nature of its mitochondrial genome. In particular, the curve does not converge on 100% genome coverage until a copy number of >100, indicating that some positions in the genome exhibit significant similarity with duplicated sequences in more than 100 other places in the genome.
(PDF)

Figure S4 **Alternative genome conformations generated by repeat-mediated recombination.** (A) A classic representation of multi-partite genome structure in plant mitochondria with a “master circle” genome conformation (left) interconverting with an alternative conformation consisting of two subcircles (right), based on recombination between a pair of direct repeats (red boxes). (B) Because of recombination, a two-copy repeat can potentially occur in any of four genomic “environments,” identified here as Reference 1 and 2 and Recombinant 1 and 2. (C) Paired-end sequencing reads that span the repeat can be used to quantify the relative abundance of these alternative conformations. Read pairs are generated by sequencing only the ends of a larger fragment from a sheared and size-selected DNA library. The solid blue lines depict read pairs that span the repeat and map consistently relative to the reference assembly with “left” and “right” ends mapping on either side of the repeat in the expected orientation and at the expected distance apart. The dashed blue lines depict read pairs that are inconsistent with the reference genome, but are consistent with one of the expected products of recombination across the shared repeat sequence. The read count data shown correspond to a 168-bp repeat pair in *S. vulgaris* Chromosome 1. In this example, there are a total of 986 (505+481) read pairs that support the reference assembly and only 20 (8+12) that support the recombinant forms. Therefore, the frequency of recombinant products associated with this repeat pair is approximately 2%.
(PDF)

Figure S5 **Relationship between length and sequence identity for repeats in** ***S. noctiflora*** **(top) and** ***S. conica*** **(bottom) mtDNA.** Each point represents a single pair of repeated sequences identified by BLAST, with the *x*-axis showing the aligned length of those sequences and the *y*-axis describing the extent of sequence similarity between the pair. Note that, in contrast to the large number of imperfect repeats (sequence identity <100%) in *S. noctiflora* and *S. conica*, all *S. latifolia* and *S. vulgaris* repeats in this size range are 100% identical or nearly so (Figure 7).
(PDF)

Figure S6 **Assays of repeat-mediated recombinational activity in** ***Silene*** **mitochondrial genomes.** (A) The left column shows the data presented in Figure 6 individually for each species (note the change in scale for each species). (B) The right column reports the analysis of the exact same repeats except in reversed orientation as a measure of the baseline level of alternative genome conformations and/or library construction artifacts in each species. Note that not all repeat pairs are shown (see Materials and Methods for filtering criteria).
(PDF)

Table S1 **Duplicate genes in** ***Silene*** **mitochondrial genomes.** Values indicate cases where more than one full-length gene or exon copy exists within the corresponding genome. Bold values indicate that the coexisting copies differ in sequence. For cases in which a mixture of identical and divergent copies exist, the total number of copies is shown in plain text and the number of unique sequences is shown parenthetically in bold. Numerous cases of duplicated gene fragments representing less than a full-length gene or exon are not reported here.
(DOC)

Table S2 **Summary of length, GC content, gene content, and GenBank accession numbers for circular chromosomes (and partially assembled genomic fragments in** ***S. conica*** **).**
(DOC)

Table S3 **Nucleotide polymorphism within** ***Silene*** **species.**
(DOC)

Table S4 **Source of** ***S. noctiflora*** **and** ***S. conica*** **populations for polymorphism analysis.**
(DOC)

Table S5 **CAPS markers used to screen for maternal inheritance of mtDNA in greenhouse crosses.**
(DOC)

## Abbreviations

CAPS: cleaved amplified polymorphic sequences
MBH: mutational burden hypothesis
mtDNA: mitochondrial DNA
