# Supplementary figures and images for: Rapid Evolution of Enormous, Multichromosomal Genomes in Flowering Plant Mitochondria with Exceptionally High Mutation Rates
Source: PLoS Biol. 2012 Jan 17;10(1):e1001241. doi: 10.1371/journal.pbio.1001241 (PMC3260318; doi:10.1371/journal.pbio.1001241)

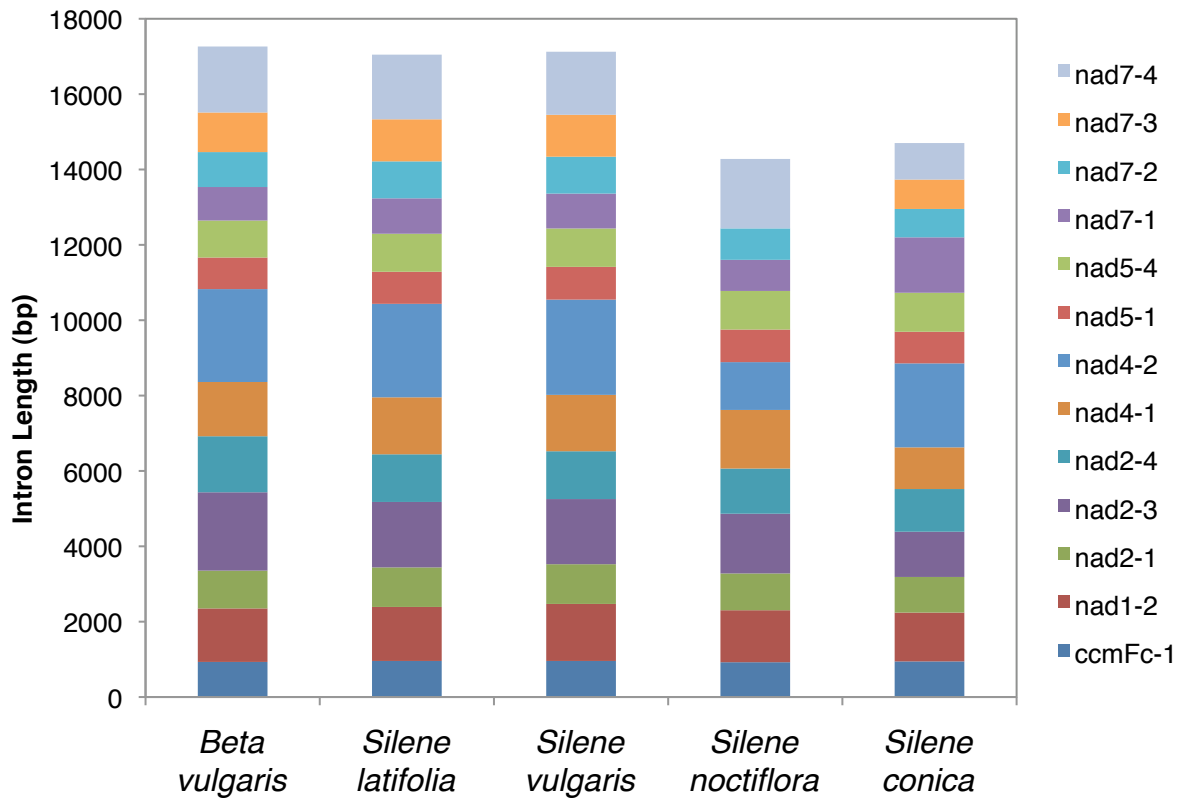

Supplement: Figure S1 — Lengths of cis -spliced introns in Silene mitochondrial genomes. (PDF) [file pbio.1001241.s001.pdf]

**A**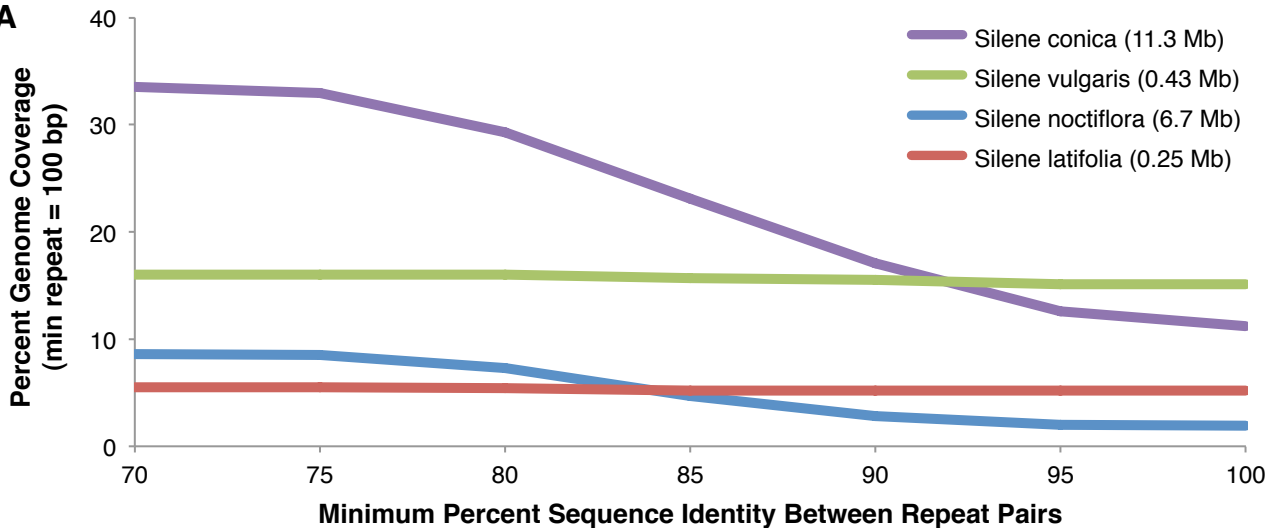**B**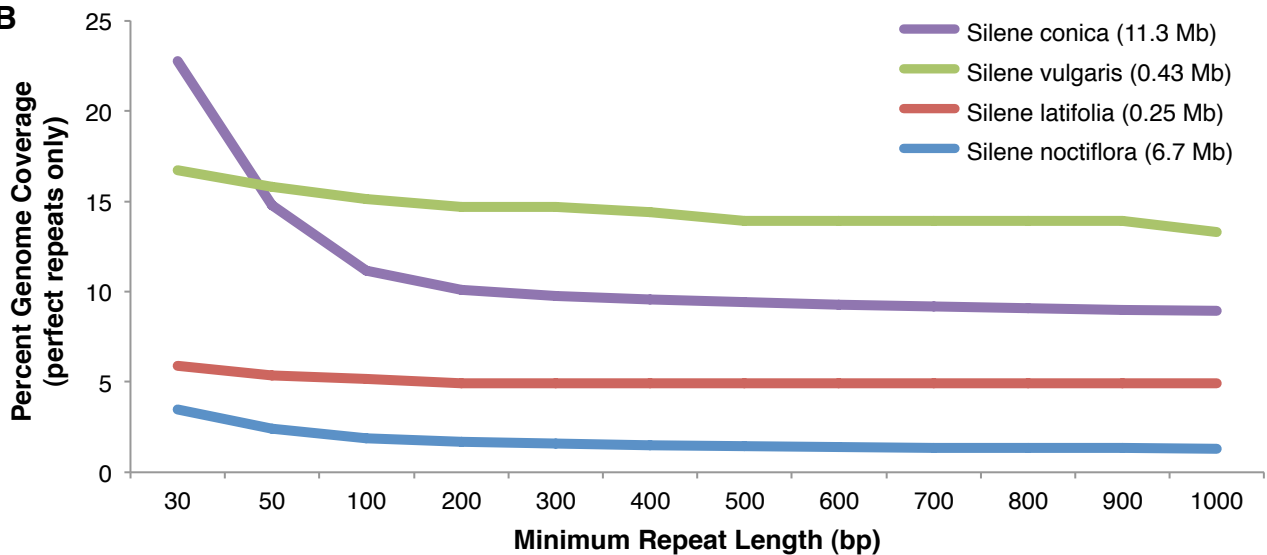

Supplement: Figure S2 — The effect of sequence identity and repeat length thresholds on estimates of repetitive content in Silene mitochondrial genomes. Perfect repeats were identified with Vmatch, and imperfect repeats were identified with BLAST (see Materials and Methods). (A) The relationship between the percent of the genome covered by repeats and the minimum percent sequence identity (based on repeats pairs of at least 100 bp in length). (B) The relationship between the percent of the genome covered and repeat length (based on perfect repeat pairs only). (PDF) [file pbio.1001241.s002.pdf]

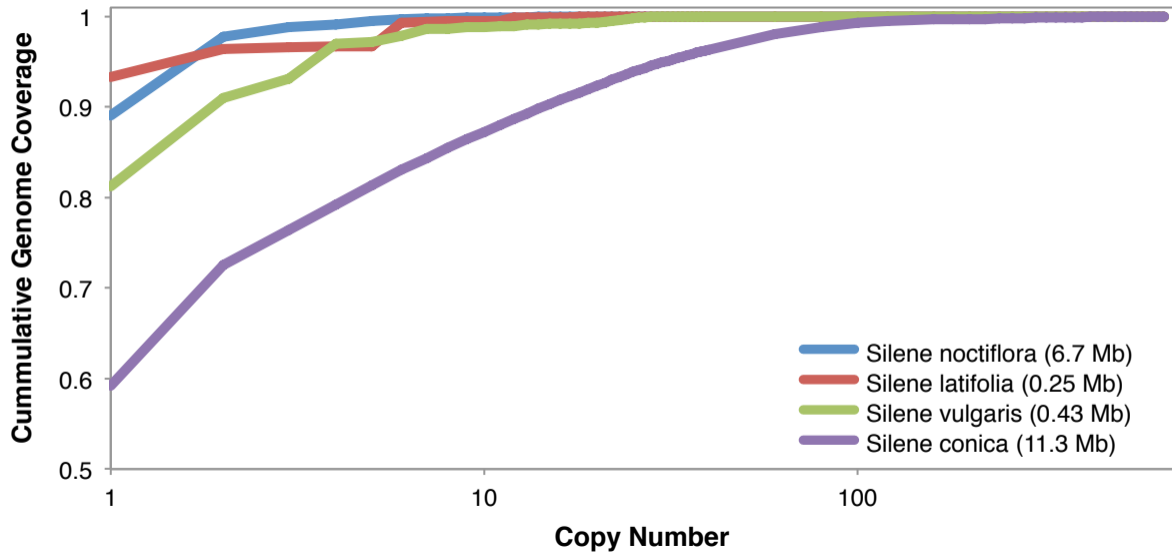

Supplement: Figure S3 — Repeat coverage depth in Silene mitochondrial genomes. For each curve, the y-intercept indicates the proportion of the mitochondrial genome that is single-copy in that species. Other points along the curve indicate the cumulative genomic coverage up to a certain repeat depth. For example, the height of the curve at a value of 10 on the x-axis indicates the fraction of the genome represented by all nucleotide positions that match nine or fewer repeats elsewhere in the genome. The lower position of the S. conica curve reflects the highly repetitive nature of its mitochondrial genome. In particular, the curve does not converge on 100% genome coverage until a copy number of >100, indicating that some positions in the genome exhibit significant similarity with duplicated sequences in more than 100 other places in the genome. (PDF) [file pbio.1001241.s003.pdf]

**A**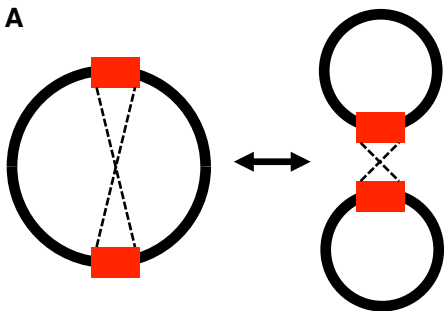**B**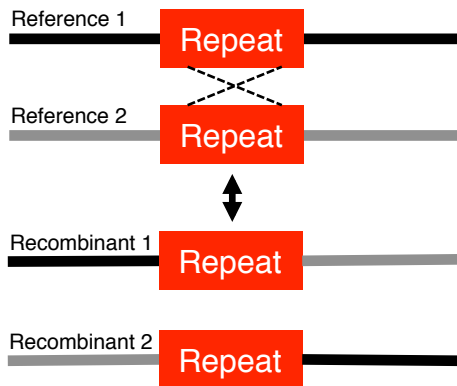**C**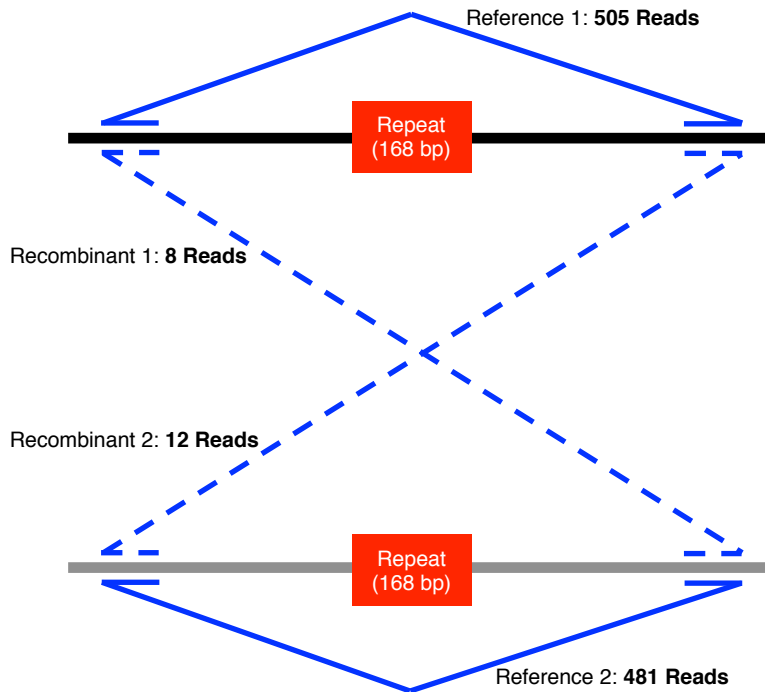

Supplement: Figure S4 — Alternative genome conformations generated by repeat-mediated recombination. (A) A classic representation of multi-partite genome structure in plant mitochondria with a “master circle” genome conformation (left) interconverting with an alternative conformation consisting of two subcircles (right), based on recombination between a pair of direct repeats (red boxes). (B) Because of recombination, a two-copy repeat can potentially occur in any of four genomic “environments,” identified here as Reference 1 and 2 and Recombinant 1 and 2. (C) Paired-end sequencing reads that span the repeat can be used to quantify the relative abundance of these alternative conformations. Read pairs are generated by sequencing only the ends of a larger fragment from a sheared and size-selected DNA library. The solid blue lines depict read pairs that span the repeat and map consistently relative to the reference assembly with “left” and “right” ends mapping on either side of the repeat in the expected orientation and at the expected distance apart. The dashed blue lines depict read pairs that are inconsistent with the reference genome, but are consistent with one of the expected products of recombination across the shared repeat sequence. The read count data shown correspond to a 168-bp repeat pair in S. vulgaris Chromosome 1. In this example, there are a total of 986 (505+481) read pairs that support the reference assembly and only 20 (8+12) that support the recombinant forms. Therefore, the frequency of recombinant products associated with this repeat pair is approximately 2%. (PDF) [file pbio.1001241.s004.pdf]

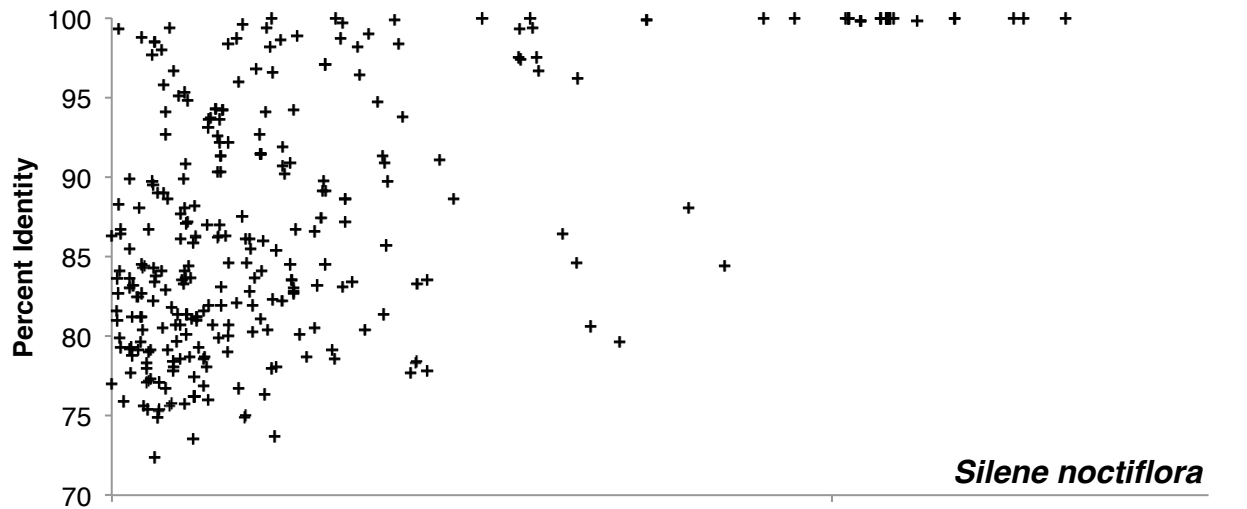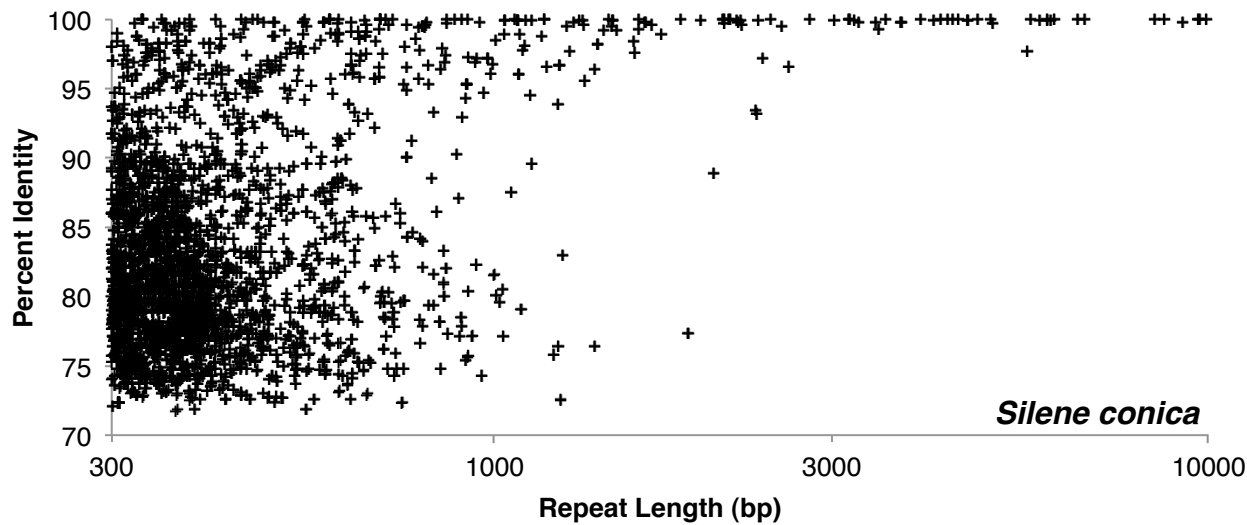

Supplement: Figure S5 — Relationship between length and sequence identity for repeats in S. noctiflora (top) and S. conica (bottom) mtDNA. Each point represents a single pair of repeated sequences identified by BLAST, with the x-axis showing the aligned length of those sequences and the y-axis describing the extent of sequence similarity between the pair. Note that, in contrast to the large number of imperfect repeats (sequence identity <100%) in S. noctiflora and S. conica, all S. latifolia and S. vulgaris repeats in this size range are 100% identical or nearly so (Figure 7). (PDF) [file pbio.1001241.s005.pdf]

### B. Reversed Orientation (Negative Control)

***Silene conica***

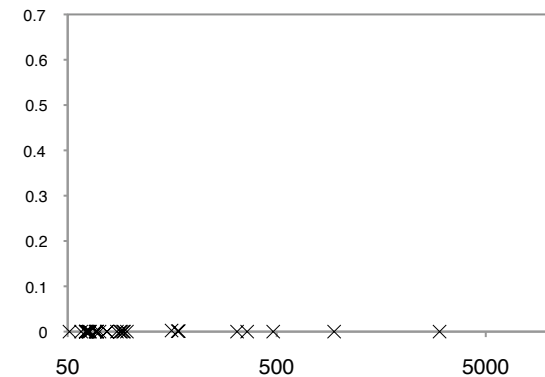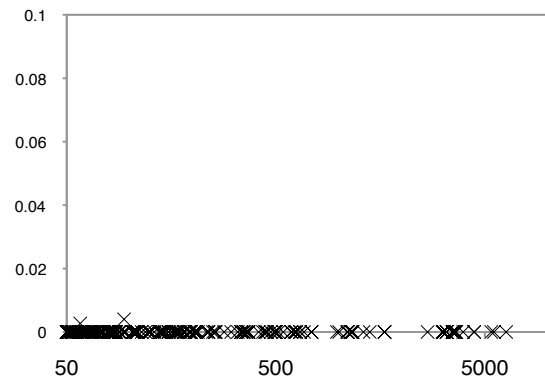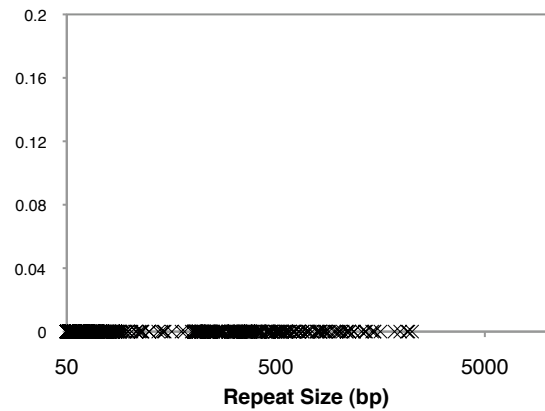

Supplement: Figure S6 — Assays of repeat-mediated recombinational activity in Silene mitochondrial genomes. (A) The left column shows the data presented in Figure 6 individually for each species (note the change in scale for each species). (B) The right column reports the analysis of the exact same repeats except in reversed orientation as a measure of the baseline level of alternative genome conformations and/or library construction artifacts in each species. Note that not all repeat pairs are shown (see Materials and Methods for filtering criteria). (PDF) [file pbio.1001241.s006.pdf]
