# Supplementary material for: Rapid Evolution of Enormous, Multichromosomal Genomes in Flowering Plant Mitochondria with Exceptionally High Mutation Rates
Source: PLoS Biol. 2012 Jan 17;10(1):e1001241. doi: 10.1371/journal.pbio.1001241 (PMC3260318; doi:10.1371/journal.pbio.1001241)
Supplement: Table S1 — Duplicate genes in Silene mitochondrial genomes. Values indicate cases where more than one full-length gene or exon copy exists within the corresponding genome. Bold values indicate that the coexisting copies differ in sequence. For cases in which a mixture of identical and divergent copies exist, the total number of copies is shown in plain text and the number of unique sequences is shown parenthetically in bold. Numerous cases of duplicated gene fragments representing less than a full-length gene or exon are not reported here. (DOC) [file pbio.1001241.s007.doc]

| **Gene** | ***Silene latifolia*** | ***Silene vulgaris*** | ***Silene noctiflora*** | ***Silene conica*** |
| --- | --- | --- | --- | --- |
|  | (0.25 Mb, 1 Chromosome) | (0.43 Mb, 4 Chromosomes) | (6.7 Mb, 59 Chromosomes) | (11.3 Mb, 128 Chromosomes) |
| *atp4* | . | . | 2 | . |
| *atp6* | . | . | . | **2** |
| *atp8* | . | . | . | 3 |
| *ccmB* | . | . | 3 | 2 |
| *ccmFc* | . | . | . | 2 |
| *cox3* | . | . | . | 3 |
| *mttB* | . | . | **2** | . |
| *nad1-exons2-3* | . | . | . | 2 |
| *nad1-exon5* | . | . | . | **2** |
| *nad2-exon1* | . | . | **4** | **.** |
| *nad2-exon2* | . | . | **2** | **.** |
| *nad3* | . | . | 3 | 4 |
| *nad4* | . | . | . | 2 |
| *nad4L* | . | . | 2 | . |
| *rpl5* | . | . | **2** | . |
| *rps13* | . | . | **2** | 3(**2**) |
| *rps3* | . | . | . | 2 |
| *rrn26* | . | . | 5 | 3 |
| *rrn18* | . | . | 5 | 4 |
| *rrn5* | **2** | . | 5 | **5** |
| *trnfM* | . | . | 5(**3**) | **3** |
| *trnI* | . | . | 5(**3**) | 3(**2**) |
