# Supplementary material for: Rapid Evolution of Enormous, Multichromosomal Genomes in Flowering Plant Mitochondria with Exceptionally High Mutation Rates
Source: PLoS Biol. 2012 Jan 17;10(1):e1001241. doi: 10.1371/journal.pbio.1001241 (PMC3260318; doi:10.1371/journal.pbio.1001241)
Supplement: Table S2 — Summary of length, GC content, gene content, and GenBank accession numbers for circular chromosomes (and partially assembled genomic fragments in S. conica ). (DOC) [file pbio.1001241.s008.doc]

| **Species** | **Chromosome No.** | **Length (bp)** | **GC Content** | **Intact Genes** | | **GenBank** | |
| --- | --- | --- | --- | --- | --- | --- | --- |
| *Silene latifolia* | 1 | 253,413 | 42.56% | 37 | | HM562727 | |
| *Silene vulgaris* | 1 | 394,403 | 41.63% | 31 | | JF750427 | |
| *Silene vulgaris* | 2 | 14,341 | 41.06% | 1 | | JF750429 | |
| *Silene vulgaris* | 3 | 12,697 | 45.86% | 4 | | JF750430 | |
| *Silene vulgaris* | 4 | 5,697 | 46.31% | 0 | | JF750428 | |
| *Silene noctiflora* | 1 | 191,963 | 40.70% | 3 | | JF750481 | |
| *Silene noctiflora* | 2 | 161,299 | 40.66% | 1 | | JF750484 | |
| *Silene noctiflora* | 3 | 153,996 | 41.17% | 1 | | JF750436 | |
| *Silene noctiflora* | 4 | 152,707 | 41.25% | 1 | | JF750485 | |
| *Silene noctiflora* | 5 | 148,612 | 41.22% | 3 | | JF750460 | |
| *Silene noctiflora* | 6 | 147,958 | 40.80% | 2 | | JF750443 | |
| *Silene noctiflora* | 7 | 146,587 | 41.03% | 2 | | JF750469 | |
| *Silene noctiflora* | 8 | 145,239 | 41.10% | 1 | | JF750478 | |
| *Silene noctiflora* | 9 | 142,006 | 40.70% | 2 | | JF750470 | |
| *Silene noctiflora* | 10 | 140,341 | 40.78% | 1 | | JF750437 | |
| *Silene noctiflora* | 11 | 138,824 | 41.08% | 1 | | JF750465 | |
| *Silene noctiflora* | 12 | 134,806 | 41.14% | 1 | | JF750433 | |
| *Silene noctiflora* | 13 | 130,152 | 40.81% | 0 | | JF750451 | |
| *Silene noctiflora* | 14 | 130,008 | 40.25% | 0 | | JF750431 | |
| *Silene noctiflora* | 15 | 129,012 | 40.81% | 0 | | JF750472 | |
| *Silene noctiflora* | 16 | 128,843 | 40.33% | 1 | | JF750453 | |
| *Silene noctiflora* | 17 | 127,930 | 40.03% | 0 | | JF750434 | |
| *Silene noctiflora* | 18 | 127,245 | 40.40% | 0 | | JF750445 | |
| *Silene noctiflora* | 19 | 127,193 | 42.28% | 1 | | JF750486 | |
| *Silene noctiflora* | 20 | 126,609 | 40.64% | 1 | | JF750456 | |
| *Silene noctiflora* | 21 | 126,452 | 40.58% | 0 | | JF750477 | |
| *Silene noctiflora* | 22 | 122,890 | 40.35% | 1 | | JF750440 | |
| *Silene noctiflora* | 23 | 122,485 | 40.60% | 1 | | JF750452 | |
| *Silene noctiflora* | 24 | 119,408 | 40.69% | 0 | | JF750448 | |
| *Silene noctiflora* | 25 | 115,341 | 40.98% | 1 | | JF750447 | |
| *Silene noctiflora* | 26 | 114,914 | 40.81% | 0 | | JF750471 | |
| *Silene noctiflora* | 27 | 113,308 | 42.15% | 2 | | JF750479 | |
| *Silene noctiflora* | 28 | 108,290 | 41.02% | 2 | | JF750462 | |
| *Silene noctiflora* | 29 | 108,152 | 41.25% | 1 | | JF750450 | |
| *Silene noctiflora* | 30 | 108,040 | 40.58% | 0 | | JF750442 | |
| *Silene noctiflora* | 31 | 107,738 | 40.08% | 0 | | JF750449 | |
| *Silene noctiflora* | 32 | 106,477 | 40.57% | 1 | | JF750466 | |
| *Silene noctiflora* | 33 | 104,288 | 40.96% | 1 | | JF750454 | |
| *Silene noctiflora* | 34 | 103,926 | 40.70% | 1 | | JF750489 | |
| *Silene noctiflora* | 35 | 103,557 | 40.05% | 0 | | JF750432 | |
| *Silene noctiflora* | 36 | 103,548 | 40.22% | 1 | | JF750435 | |
| *Silene noctiflora* | 37 | 103,320 | 40.70% | 0 | | JF750458 | |
| *Silene noctiflora* | 38 | 102,347 | 40.14% | 2 | | JF750444 | |
| *Silene noctiflora* | 39 | 100,876 | 40.46% | 1 | | JF750467 | |
| *Silene noctiflora* | 40 | 100,579 | 41.45% | 3 | | JF750459 | |
| *Silene noctiflora* | 41 | 100,078 | 40.47% | 0 | | JF750438 | |
| *Silene noctiflora* | 42 | 98,550 | 40.59% | 1 | | JF750464 | |
| *Silene noctiflora* | 43 | 97,627 | 40.50% | 0 | | JF750439 | |
| *Silene noctiflora* | 44 | 96,564 | 41.12% | 1 | | JF750475 | |
| *Silene noctiflora* | 45 | 96,233 | 40.71% | 0 | | JF750480 | |
| *Silene noctiflora* | 46 | 95,084 | 40.92% | 0 | | JF750441 | |
| *Silene noctiflora* | 47 | 94,621 | 41.15% | 2 | | JF750446 | |
| *Silene noctiflora* | 48 | 94,201 | 39.91% | 0 | | JF750487 | |
| *Silene noctiflora* | 49 | 92,946 | 41.03% | 1 | | JF750457 | |
| *Silene noctiflora* | 50 | 92,480 | 40.75% | 3 | | JF750463 | |
| *Silene noctiflora* | 51 | 92,366 | 40.92% | 0 | | JF750476 | |
| *Silene noctiflora* | 52 | 91,804 | 41.15% | 1 | | JF750455 | |
| *Silene noctiflora* | 53 | 91,595 | 40.79% | 2 | | JF750474 | |
| *Silene noctiflora* | 54 | 89,951 | 40.93% | 0 | | JF750488 | |
| *Silene noctiflora* | 55 | 86,782 | 39.77% | 1 | | JF750473 | |
| *Silene noctiflora* | 56 | 81,416 | 40.97% | 3 | | JF750483 | |
| *Silene noctiflora* | 57 | 74,922 | 40.57% | 2 | | JF750461 | |
| *Silene noctiflora* | 58 | 67,018 | 40.71% | 0 | | JF750468 | |
| *Silene noctiflora* | 59 | 66,365 | 40.66% | 3 | | JF750482 | |
| *Silene conica* | 1 | 163,071 | 43.08% | 0 | | JF750534 | |
| *Silene conica* | 2 | 155,921 | 42.47% | 1 | | JF750515 | |
| *Silene conica* | 3 | 151,014 | 43.56% | 1 | | JF750506 | |
| *Silene conica* | 4 | 149,089 | 43.15% | 1 | | JF750505 | |
| *Silene conica* | 5 | 147,648 | 42.60% | 0 | | JF750497 | |
| *Silene conica* | 6 | 142,373 | 43.31% | 0 | | JF750508 | |
| *Silene conica* | 7 | 137,495 | 43.66% | 1 | | JF750520 | |
| *Silene conica* | 8 | 130,956 | 43.51% | 0 | | JF750557 | |
| *Silene conica* | 9 | 127,523 | 43.15% | 1 | | JF750503 | |
| *Silene conica* | 10 | 125,751 | 42.93% | 0 | | JF750563 | |
| *Silene conica* | 11 | 125,117 | 43.06% | 1 | | JF750512 | |
| *Silene conica* | 12 | 120,801 | 43.56% | 3 | | JF750511 | |
| *Silene conica* | 13 | 119,487 | 42.72% | 0 | | JF750517 | |
| *Silene conica* | 14 | 119,001 | 43.51% | 3 | | JF750513 | |
| *Silene conica* | 15 | 118,929 | 42.89% | 0 | | JF750558 | |
| *Silene conica* | 16 | 117,607 | 43.37% | 1 | | JF750516 | |
| *Silene conica* | 17 | 115,763 | 42.93% | 0 | | JF750593 | |
| *Silene conica* | 18 | 114,589 | 43.12% | 1 | | JF750518 | |
| *Silene conica* | 19 | 110,652 | 43.59% | 3 | | JF750578 | |
| *Silene conica* | 20 | 110,427 | 42.60% | 1 | | JF750519 | |
| *Silene conica* | 21 | 108,941 | 42.98% | 0 | | JF750555 | |
| *Silene conica* | 22 | 108,940 | 42.66% | 0 | | JF750536 | |
| *Silene conica* | 23 | 107,216 | 43.61% | 3 | | JF750510 | |
| *Silene conica* | 24 | 106,709 | 42.67% | 0 | | JF750526 | |
| *Silene conica* | 25 | 105,730 | 42.95% | 0 | | JF750532 | |
| *Silene conica* | 26 | 105,655 | 43.24% | 2 | | JF750531 | |
| *Silene conica* | 27 | 105,520 | 43.78% | 0 | | JF750504 | |
| *Silene conica* | 28 | 103,419 | 43.08% | 1 | | JF750501 | |
| *Silene conica* | 29 | 103,231 | 43.42% | 1 | | JF750599 | |
| *Silene conica* | 30 | 103,102 | 42.75% | 0 | | JF750527 | |
| *Silene conica* | 31 | 103,003 | 42.35% | 0 | | JF750494 | |
| *Silene conica* | 32 | 102,316 | 43.32% | 2 | | JF750587 | |
| *Silene conica* | 33 | 102,020 | 43.69% | 0 | | JF750588 | |
| *Silene conica* | 34 | 101,608 | 43.27% | 0 | | JF750535 | |
| *Silene conica* | 35 | 101,353 | 42.93% | 1 | | JF750547 | |
| *Silene conica* | 36 | 100,148 | 43.41% | 1 | | JF750528 | |
| *Silene conica* | 37 | 99,951 | 43.88% | 0 | | JF750493 | |
| *Silene conica* | 38 | 99,512 | 43.73% | 0 | | JF750533 | |
| *Silene conica* | 39 | 98,308 | 42.73% | 2 | | JF750529 | |
| *Silene conica* | 40 | 98,068 | 42.61% | 0 | | JF750545 | |
| *Silene conica* | 41 | 97,429 | 42.93% | 2 | | JF750546 | |
| *Silene conica* | 42 | 97,283 | 42.49% | 0 | | JF750548 | |
| *Silene conica* | 43 | 97,269 | 43.10% | 0 | | JF750583 | |
| *Silene conica* | 44 | 97,111 | 43.83% | 0 | | JF750522 | |
| *Silene conica* | 45 | 96,762 | 42.38% | 0 | | JF750596 | |
| *Silene conica* | 46^a^ | 96,395 | 43.30% | 0 | | JF750626 | |
| *Silene conica* | 47 | 93,489 | 43.75% | 1 | | JF750614 | |
| *Silene conica* | 48 | 93,235 | 42.79% | 0 | | JF750530 | |
| *Silene conica* | 49 | 92,438 | 43.65% | 2 | | JF750624 | |
| *Silene conica* | 50 | 91,619 | 43.22% | 0 | | JF750538 | |
| *Silene conica* | 51 | 91,401 | 43.21% | 1 | | JF750597 | |
| *Silene conica* | 52 | 88,472 | 43.12% | 1 | | JF750564 | |
| *Silene conica* | 53 | 88,260 | 42.84% | 1 | | JF750543 | |
| *Silene conica* | 54 | 87,616 | 43.50% | 0 | | JF750542 | |
| *Silene conica* | 55 | 86,501 | 43.40% | 0 | | JF750521 | |
| *Silene conica* | 56 | 85,606 | 42.75% | 1 | | JF750523 | |
| *Silene conica* | 57 | 85,117 | 43.44% | 1 | | JF750514 | |
| *Silene conica* | 58^a^ | 85,095 | 43.21% | 1 | | JF750627 | |
| *Silene conica* | 59 | 84,942 | 42.64% | 1 | | JF750495 | |
| *Silene conica* | 60 | 84,201 | 43.09% | 1 | | JF750539 | |
| *Silene conica* | 61 | 83,288 | 42.77% | 2 | | JF750610 | |
| *Silene conica* | 62 | 82,551 | 43.30% | 0 | | JF750585 | |
| *Silene conica* | 63 | 81,689 | 43.12% | 1 | | JF750544 | |
| *Silene conica* | 64 | 81,671 | 43.07% | 1 | | JF750507 | |
| *Silene conica* | 65 | 81,294 | 42.56% | 0 | | JF750569 | |
| *Silene conica* | 66 | 81,243 | 43.19% | 0 | | JF750540 | |
| *Silene conica* | 67 | 80,675 | 42.57% | 0 | | JF750541 | |
| *Silene conica* | 68 | 79,841 | 43.68% | 0 | | JF750574 | |
| *Silene conica* | 69 | 79,368 | 43.98% | 0 | | JF750572 | |
| *Silene conica* | 70 | 79,279 | 43.09% | 0 | | JF750525 | |
| *Silene conica* | 71 | 78,978 | 42.85% | 0 | | JF750576 | |
| *Silene conica* | 72 | 78,953 | 43.18% | 1 | | JF750591 | |
| *Silene conica* | 73 | 78,367 | 43.70% | 0 | | JF750549 | |
| *Silene conica* | 74 | 77,721 | 42.70% | 0 | | JF750561 | |
| *Silene conica* | 75^a^ | 77,256 | 42.85% | 0 | | JF750628 | |
| *Silene conica* | 76 | 77,149 | 43.58% | 0 | | JF750589 | |
| *Silene conica* | 77 | 76,904 | 42.63% | 0 | | JF750550 | |
| *Silene conica* | 78 | 76,619 | 43.14% | 0 | | JF750556 | |
| *Silene conica* | 79 | 76,441 | 43.50% | 0 | | JF750559 | |
| *Silene conica* | 80 | 76,039 | 42.82% | 0 | | JF750551 | |
| *Silene conica* | 81 | 75,310 | 44.06% | 0 | | JF750605 | |
| *Silene conica* | 82 | 74,810 | 43.34% | 0 | | JF750552 | |
| *Silene conica* | 83 | 74,506 | 43.02% | 0 | | JF750562 | |
| *Silene conica* | 84 | 73,936 | 43.40% | 0 | | JF750553 | |
| *Silene conica* | 85 | 73,670 | 43.66% | 0 | | JF750566 | |
| *Silene conica* | 86 | 73,557 | 42.77% | 1 | | JF750554 | |
| *Silene conica* | 87 | 73,226 | 42.98% | 0 | | JF750490 | |
| *Silene conica* | 88 | 71,828 | 42.74% | 0 | | JF750567 | |
| *Silene conica* | 89 | 70,246 | 42.63% | 0 | | JF750601 | |
| *Silene conica* | 90 | 69,453 | 43.58% | 0 | | JF750598 | |
| *Silene conica* | 91 | 69,443 | 43.85% | 2 | | JF750602 | |
| *Silene conica* | 92 | 68,046 | 43.32% | 0 | | JF750586 | |
| *Silene conica* | 93 | 67,617 | 42.47% | 0 | | JF750491 | |
| *Silene conica* | 94 | 67,525 | 43.40% | 2 | | JF750524 | |
| *Silene conica* | 95 | 67,127 | 43.57% | 0 | | JF750560 | |
| *Silene conica* | 96 | 66,830 | 43.66% | 3 | | JF750612 | |
| *Silene conica* | 97 | 66,402 | 43.30% | 0 | | JF750608 | |
| *Silene conica* | 98 | 65,772 | 43.15% | 0 | | JF750565 | |
| *Silene conica* | 99 | 65,543 | 42.98% | 1 | | JF750590 | |
| *Silene conica* | 100 | 65,424 | 42.94% | 0 | | JF750604 | |
| *Silene conica* | 101 | 65,272 | 43.14% | 2 | | JF750606 | |
| *Silene conica* | 102 | 65,085 | 42.82% | 0 | | JF750499 | |
| *Silene conica* | 103 | 64,608 | 43.08% | 0 | | JF750571 | |
| *Silene conica* | 104 | 64,281 | 42.56% | 0 | | JF750575 | |
| *Silene conica* | 105 | 63,852 | 42.89% | 0 | | JF750603 | |
| *Silene conica* | 106 | 63,308 | 43.03% | 0 | | JF750502 | |
| *Silene conica* | 107 | 63,002 | 42.39% | 0 | | JF750496 | |
| *Silene conica* | 108 | 62,749 | 43.19% | 2 | | JF750595 | |
| *Silene conica* | 109 | 62,571 | 42.59% | 0 | | JF750568 | |
| *Silene conica* | 110 | 62,405 | 42.50% | 0 | | JF750492 | |
| *Silene conica* | 111 | 60,812 | 42.58% | 0 | | JF750498 | |
| *Silene conica* | 112 | 60,476 | 43.99% | 0 | | JF750537 | |
| *Silene conica* | 113 | 60,079 | 42.92% | 0 | | JF750580 | |
| *Silene conica* | 114 | 59,915 | 43.06% | 0 | | JF750570 | |
| *Silene conica* | 115 | 59,463 | 42.63% | 0 | | JF750509 | |
| *Silene conica* | 116 | 58,995 | 43.34% | 0 | | JF750615 | |
| *Silene conica* | 117 | 58,600 | 43.17% | 0 | | JF750592 | |
| *Silene conica* | 118 | 58,548 | 43.27% | 0 | | JF750609 | |
| *Silene conica* | 119 | 57,218 | 43.19% | 0 | | JF750579 | |
| *Silene conica* | 120 | 55,005 | 43.15% | 0 | | JF750594 | |
| *Silene conica* | 121 | 54,881 | 43.55% | 0 | | JF750611 | |
| *Silene conica* | 122 | 54,719 | 43.14% | 0 | | JF750581 | |
| *Silene conica* | 123 | 54,696 | 43.46% | 2 | | JF750582 | |
| *Silene conica* | 124 | 54,650 | 42.92% | 0 | | JF750577 | |
| *Silene conica* | 125 | 52,701 | 43.29% | 0 | | JF750573 | |
| *Silene conica* | 126 | 52,589 | 42.33% | 0 | | JF750607 | |
| *Silene conica* | 127 | 46,047 | 42.39% | 0 | | JF750584 | |
| *Silene conica* | 128 | 43,958 | 42.46% | 0 | | JF750600 | |
| *Silene conica* | Fragment_01^a,b^ | 52,090 | 43.02% | 0 | | JF750629 | |
| *Silene conica* | Fragment_02^b^ | 51,272 | 42.85% | 0 | | JF750613 | |
| *Silene conica* | Fragment_03^b^ | 44,768 | 43.08% | 0 | | JF750500 | |
| *Silene conica* | Fragment_04^b^ | 40,160 | 42.70% | 0 | | JF750616 | |
| *Silene conica* | Fragment_05^b^ | 4,346 | 44.91% | 0 | | JF750620 | |
| *Silene conica* | Fragment_06^b^ | 4,039 | 46.50% | 0 | | JF750621 | |
| *Silene conica* | Fragment_07^c^ | 3,765 | 48.76% | 0 | | JF750625 | |
| *Silene conica* | Fragment_08^b^ | 1,959 | 46.66% | 0 | | JF750623 | |
| *Silene conica* | Fragment_09^b^ | 576 | 54.17% | 0 | | JF750622 | |
| *Silene conica* | Fragment_10^b^ | 503 | 39.96% | 0 | | JF750619 | |
| *Silene conica* | Fragment_11^b^ | 353 | 44.19% | 0 | | JF750618 | |
| *Silene conica* | Fragment_12^b^ | 288 | 54.17% | 0 | | JF750617 | |
| ^a^Sequence broken into two pieces because of assembly gaps. | | | | |  | |  |
| ^b^Unassembled fragment with alternative connections to other parts of the genome. Not circular mapping. | | | | | | | |
| ^c^Uncharacterized high-copy element. | |  |  |  | |  | |
