# Supplementary material for: Rapid Evolution of Enormous, Multichromosomal Genomes in Flowering Plant Mitochondria with Exceptionally High Mutation Rates
Source: PLoS Biol. 2012 Jan 17;10(1):e1001241. doi: 10.1371/journal.pbio.1001241 (PMC3260318; doi:10.1371/journal.pbio.1001241)
Supplement: Table S3 — Nucleotide polymorphism within Silene species. (DOC) [file pbio.1001241.s009.doc]

| **Table S3.** Nucleotide polymorphism within *Silene* species | | | | |  |  |  | |  |  |  |  |
| --- | --- | --- | --- | --- | --- | --- | --- | --- | --- | --- | --- | --- |
|  |  |  | ***Silene latifolia* (*n* = 28)** | | | |  | | ***Silene vulgaris* (*n* = 40)** | | | |
| **Mitochondrial** | **Length (nt)** |  | ***S*** | ***Θ_W_* (95% CI)** | ***π_S_*** | ***π_N_*** |  | | ***S*** | ***Θ_W_* (95% CI)** | ***π_S_*** | ***π_N_*** |
| *atp1* | 951 |  | 4 | 0.0011 (0.0003-0.0032) | 0.003 | 0.001 |  | | 24 | 0.0062 (0.0029-0.0120) | 0.023 | 0.001 |
| *atp4* | 246-282 |  | 1 | 0.0010 (0.0001-0.0049) | 0.000 | 0.002 |  | | 0 | 0.0000 (0.0000-0.0018) | 0.000 | 0.000 |
| *atp6* | 282 |  | 1 | 0.0009 (0.0001-0.0047) | 0.002 | 0.000 |  | | 2 | 0.0017 (0.0002-0.0061) | 0.005 | 0.000 |
| *cox3* | 597 |  | 4 | 0.0018 (0.0004-0.0051) | 0.001 | 0.002 |  | | 5 | 0.0020 (0.0006-0.0053) | 0.000 | 0.002 |
| *nad9* | 393 |  | 4 | 0.0027 (0.0006-0.0078) | 0.006 | 0.001 |  | | 1 | 0.0006 (0.0000-0.0030) | 0.000 | 0.001 |
| *nad4L-atp4* (intergenic) | 136-513 |  | 1 | 0.0019 (0.0001-0.0096) | 0.001 | -- |  | | 4 | 0.0068 (0.0018-0.0193) | 0.004 | -- |
| Total (coding only) | 2469-2505 |  | 14 | 0.0015 (0.0006-0.0032) | 0.002 | 0.001 |  | | 32 | 0.0032 (0.0016-0.0059) | 0.010 | 0.001 |
|  |  |  |  |  |  |  |  | |  |  |  |  |
| **Plastid** |  |  |  |  |  |  |  | |  |  |  |  |
| *trnL* (intronic) | 384-509 |  | 7 | 0.0038 (0.0013-0.0094) | 0.001 | -- |  | | 7 | 0.0034 (0.0012-0.0081) | 0.001 | -- |
|  |  |  |  |  |  |  |  | |  |  |  |  |
| **Nuclear** |  |  |  |  |  |  |  | |  |  |  |  |
| fructose-2,6-bisphosphatase (X4/XY4) | 528 |  | 41 | 0.0212 (0.0102-0.0409) | 0.086 | 0.010 |  | | 44 | 0.0208 (0.0106-0.0379) | 0.042 | 0.004 |
|  |  |  |  |  |  |  | |  |  |  |  |
|  |  |  |  |  |  |  |  | |  |  |  |  |
|  |  |  | ***Silene conica* (*n* = 5)** | | | |  | | ***Silene noctiflora* (*n* = 9)** | | | |
| **Mitochondrial** | **Length (nt)** |  | ***S*** | ***Θ_W_* (95% CI)** | ***π_S_*** | ***π_N_*** |  | | ***S*** | ***Θ_W_* (95% CI)** | ***π_S_*** | ***π_N_*** |
| *atp1* | 951 |  | 5 | 0.0026 (0.0006-0.0115) | 0.008 | 0.001 |  | | 0 | 0.0000 (0.0000-0.0009) | 0.000 | 0.000 |
| *atp4* | 246-282 |  | 7 | 0.0124 (0.0032-0.0514) | 0.038 | 0.004 |  | | 0 | 0.0000 (0.0000-0.0033) | 0.000 | 0.000 |
| *atp6* | 282 |  | 7 | 0.0124 (0.0032-0.0514) | 0.038 | 0.002 |  | | 0 | 0.0000 (0.0000-0.0032) | 0.000 | 0.000 |
| *cox3* | 597 |  | 2 | 0.0017 (0.0002-0.0091) | 0.003 | 0.001 |  | | 0 | 0.0000 (0.0000-0.0015) | 0.000 | 0.000 |
| *nad9* | 393 |  | 4 | 0.0051 (0.0010-0.0232) | 0.016 | 0.002 |  | | 0 | 0.0000 (0.0000-0.0023) | 0.000 | 0.000 |
| *nad4L-atp4* (intergenic) | 136-513 |  | 1 | 0.0035 (0.0001-0.0262) | 0.004 | -- |  | | 0 | 0.0000 (0.0000-0.0065) | 0.000 | -- |
| Total (coding only) | 2469-2505 |  | 25 | 0.0050 (0.0017-0.0186) | 0.015 | 0.002 |  | | 0 | 0.0000 (0.0000-0.0004) | 0.000 | 0.000 |
|  |  |  |  |  |  |  |  | |  |  |  |  |
| **Plastid** |  |  |  |  |  |  |  | |  |  |  |  |
| *trnL* (intronic) | 384-509 |  | 0 | 0.0000 (0.0000-0.0026) | 0.000 | -- |  | | 0 | 0.0000 (0.0000-0.0021) | 0.000 | -- |
|  |  |  |  |  |  |  |  | |  |  |  |  |
| **Nuclear** |  |  |  |  |  |  |  | |  |  |  |  |
| fructose-2,6-bisphosphatase (X4/XY4) | 528 |  | 14 | 0.0133 (0.0042-0.0511) | 0.047 | 0.001 |  | | 0 | 0.0000 (0.0000-0.0017) | 0.000 | 0.000 |
|  |  |  |  |  |  |  | |  |  |  |  |
|  |  |  |  |  |  |  |  | |  |  |  |  |
| *S* = Number of segregating sites | |  |  |  |  |  |  | |  |  |  |  |
| *Θ_W_ =* Watterson's *Θ* per bp |  |  |  |  |  |  |  | |  |  |  |  |
| *π_S_* = Synonymous (or non-coding in the case of introns and intergenic sequences) nucleotide diversity | | | | | | | |  | |  |  |  |
| *π_N_* = Non-synonymous nucleotide diversity | |  |  |  |  |  |  | |  |  |  |  |
| *n* = Number of sampled populations (one individual per population) | | | | |  |  |  | |  |  |  |  |
