# Supplementary material for: Rapid Evolution of Enormous, Multichromosomal Genomes in Flowering Plant Mitochondria with Exceptionally High Mutation Rates
Source: PLoS Biol. 2012 Jan 17;10(1):e1001241. doi: 10.1371/journal.pbio.1001241 (PMC3260318; doi:10.1371/journal.pbio.1001241)
Supplement: Table S4 — Source of S. noctiflora and S. conica populations for polymorphism analysis. (DOC) [file pbio.1001241.s010.doc]

|  | **Population Code** | **Seed Collection Location/Source** |
| --- | --- | --- |
| ***Silene noctiflora*** | OSR (genome) | Giles County, VA, USA |
|  | BDA | Budapest, Hungary |
|  | BRP | Nelson County, VA, USA |
|  | BWT | Tübingen, Germany |
|  | OPL | Opole, Poland |
|  | PKC | Přední Kopanina, Czech Republic |
|  | SGH | Albemarle County, VA, USA (tissue sample only) |
|  | TTP | South River, ON, Canada |
|  | UMN | Minneapolis, MN, USA (tissue sample only) |
|  |  |  |
| ***Silene conica*** | ABR (genome) | Abruzzo, Italy (provided by M. Hood; collected by F. Conti) |
|  | BOX | Mount Kitheron, Nomos Viotias, Greece (collected by B. Oxelman) |
|  | FBG | Frankfurt Botanical Garden, Germany (provided by L. Gimenez) |
|  | KGA | Wroclaw, Poland (provided by Kew Gardens) |
|  | KGB | Norfolk, England (provided by Kew Gardens) |
