# Supplementary material for: Rapid Evolution of Enormous, Multichromosomal Genomes in Flowering Plant Mitochondria with Exceptionally High Mutation Rates
Source: PLoS Biol. 2012 Jan 17;10(1):e1001241. doi: 10.1371/journal.pbio.1001241 (PMC3260318; doi:10.1371/journal.pbio.1001241)
Supplement: Table S5 — CAPS markers used to screen for maternal inheritance of mtDNA in greenhouse crosses. (DOC) [file pbio.1001241.s011.doc]

| **Species** | **Gene** | **Position** | **SNP** | **Enzyme** | **Primers^a^** |
| --- | --- | --- | --- | --- | --- |
| *Silene conica* | *atp1* | 408 | A/G | MspI | F-GAGTSRTCTCAGTTGGAGATG |
|  |  |  |  |  | R-CTTSTTTCATAGCTTTCAACTG |
| *Silene latifolia* | *nad9* | 207 | A/C | HindIII | F-TTGTGCTTTCTCAAATTGCATACC |
|  |  |  |  |  | R-CGAGTACTCAGTAAATTATAGACCACTTCAAA**G**CT |
| *Silene noctiflora* | *cox1* | 1377 | A/G | BamHI | F-CCCAATGCATTTCTTAGGACTTG |
|  |  |  |  |  | R-AAGAAAGCCGCTATTCCCACTACGGAGATATA**G**GA |
| ^a^Nucleotides shown in bold represent mismatches in the primer sequence that were introduced in order to generate a polymorphic restriction site | | | | | |
